# GATA factor TRPS1, a new DNA repair protein, cooperates with reversible PARylation to promote chemoresistance in patients with breast cancer

**DOI:** 10.1016/j.jbc.2024.107780

**Published:** 2024-09-12

**Authors:** Jun Zhang, Yatao Chen, Xue Gong, Yongfeng Yang, Yun Gu, Ling Huang, Jianfeng Fu, Menglu Zhao, Yehong Huang, Lulu Li, Wenzhuo Liu, Yajie Wan, Xilin He, Zhifang Ma, Weiyong Zhao, Meng Zhang, Tao Tang, Yuzhi Wang, Jean Paul Thiery, Xiaofeng Zheng, Liming Chen

**Affiliations:** 1 Department of Biochemistry, School of Life Sciences, Nanjing Normal University, Nanjing, China; 2 Women's Hospital of Nanjing Medical University, Nanjing Women and Children’s Healthcare Hospital, Nanjing, China; 3 State Key Lab of Protein and Plant Gene Research, Department of Biochemistry and Molecular Biology, School of Life Sciences, Peking University, Beijing, China; 4 Department of Radiation Oncology, Affiliated Hospital of Integrated Traditional Chinese and Western Medicine, Nanjing University of Chinese Medicine, Nanjing, China; 5 Institute of Molecular and Cell Biology, A∗STAR, Singapore; 6 Jiangsu Institute of Cancer Research, Jiangsu Cancer Hospital, the Affiliated Cancer Hospital of Nanjing Medical University, Nanjing, China

**Keywords:** breast cancer, chemotherapy, DNA damage repair, TRPS1, PARylation

## Abstract

Resistance to DNA-damaging agents is a major unsolved challenge for breast cancer patients undergoing chemotherapy. Here, we show that elevated expression of transcriptional repressor GATA binding 1 (TRPS1) is associated with lower drug sensitivity, reduced response rate, and poor prognosis in chemotherapy-treated breast cancer patients. Mechanistically, elevated TRPS1 expression promotes hyperactivity of DNA damage repair (DDR) in breast cancer cells. We provide evidence that TRPS1 dynamically localizes to DNA breaks in a Ku70-and Ku80-dependent manner and that TRPS1 is a new member of the DDR protein family. We also discover that the dynamics of TRPS1 assembly at DNA breaks is regulated by its reversible PARylation in the DDR, and that mutations of the PARylation sites on TRPS1 lead to increased sensitivity to chemotherapeutic drugs. Taken together, our findings provide new mechanistic insights into the DDR and chemoresistance in breast cancer patients and identify TRPS1 as a critical DDR protein. TRPS1 may also be considered as a target to improve chemo-sensitization strategies and, consequently, clinical outcomes for breast cancer patients.

Breast cancer (BC) is the most common cancer in women (1). Patients with breast cancer are often treated with DNA-damaging agents as part of chemotherapeutic regimens, which are aimed at reducing tumor growth and relieving pain (2). However, many patients with BC exhibit resistance to DNA-damaging agents, and this lack of response remains a major unresolved issue. As such, a thorough understanding of the DNA repair process is required to predict response to chemotherapy and to develop novel therapeutic strategies (3).

Hyperactivity in DNA damage repair (DDR) pathways is routinely observed in chemo-insensitive cancer cells (4), with increased expression of DNA repair proteins (DRPs) considered a key molecular event (3). However, although numerous overexpressed proteins have been identified with pathogenic behaviors in BC cells (5, 6, 7, 8, 9, 10), DRPs have yet to be linked with BC and chemoresistance.

Transcriptional repressor GATA binding 1 (TRPS1), an atypical GATA transcription factor, was first reported in 2005 to be overexpressed in BC cells in >90% of patients (11). In contrast to other GATA proteins, TRPS1 contains only one GATA domain, lying adjacent to its C-terminal domains (CTD) and N-terminal domains (NTD) (12). We and others have confirmed that TRPS1 is frequently overexpressed in BC cells, and plays an important role in BC pathogenesis by repressing the expression of various genes (13, 14, 15, 16, 17, 18, 19, 20). However, it remains to be determined whether and how TRPS1 contributes to chemotherapy failure in BC patients.

In this study, we found that elevated TRPS1 is strongly associated with resistance in BC patients undergoing chemotherapy. Through functional and mechanistic studies, we show that TRPS1 facilitates the DDR, thereby protecting BC cells from DNA-damaging drugs. In the present work, TRPS1 is found to act as a DRP that promotes the hyperactivity of the DDR in BC cells. As a DRP, TRPS1 directly and dynamically localizes to DNA breaks in DDR in a Ku-dependent manner, where reversible PARylation events in DDR play important regulatory roles. Collectively, our findings suggest that TRPS1, with its newly identified DDR function in this study, can be considered a useful marker for predicting the response of BC patients to chemotherapy. TRPS1 can also be considered a promising druggable target for the development of new chemo-sensitizing drugs.

## Results

### Elevated TRPS1 reduces BC cell chemosensitivity

**Figure 1:**
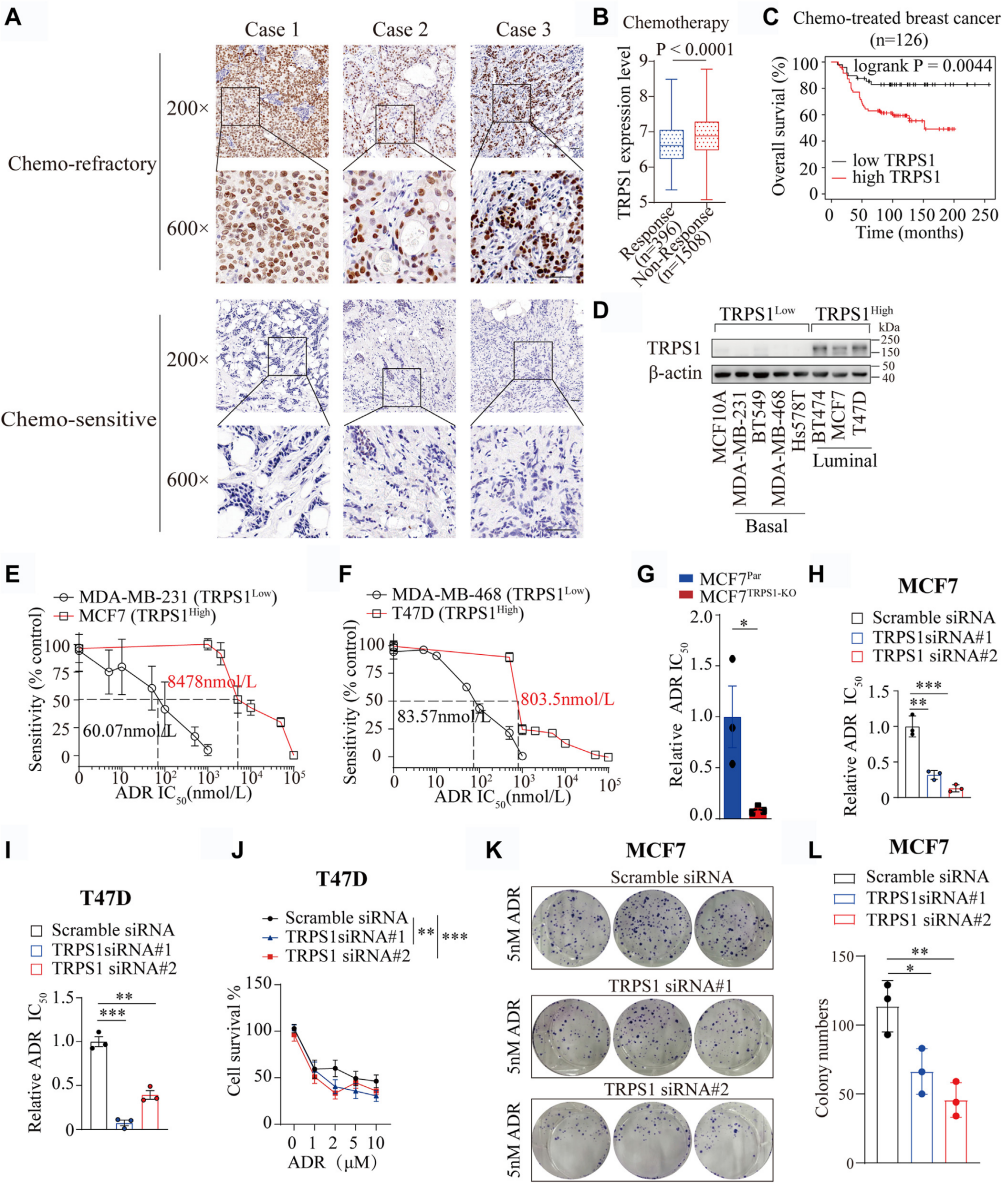
**Elevated TRPS1 promotes chemoresistance in BC.***A*, representative images of immunohistochemical staining for TRPS1 in human breast tumor tissue. Brown staining indicates positive immunoreactivity. Scale bar, 50 μm. *B*, box-and-whisker plot showing that patients with BC who do not respond to chemotherapy exhibit higher TRPS1 expression levels than those who do respond. *C*, survival analysis showing that high TRPS1 protein level is associated with poor overall survival in BC patients treated with chemotherapy. The analysis is based on published data analyzed by the KM-plotter. *D*, representative Western blot image showing TRPS1 expression levels in a panel of BC cell lines, which are classified as TRPS1^high^ (MCF7, T47D, and BT474) and TRPS1^low^ (MDA-MB-231, MDA-MB-468, BT549, and Hs578T) based on endogenous TRPS1 expression levels. *E*, MCF7 cells and MDA-MB-231 cells were treated with ADR at the indicated concentration. The ADR sensitivity was examined by cell viability assay 2 days after ADR treatment, and the results show that TRPS1^high^ BC cells are much less sensitive to ADR than TRPS1^low^ BC cells. *F*, T47D cells and MDA-MB-468 cells were treated with ADR at the indicated concentration. The ADR sensitivity was examined by cell viability assay 2 days after ADR treatment, and the results show TRPS1^high^ BC cells are much less sensitive to ADR than TRPS1^low^ BC cells. *G*, MCF7^Par^ and MCF7^TRPS1-KO^ cells were treated with different concentrations of ADR and the cell viability was examined 2 days after ADR treatment. The IC50 value was calculated using GraphPad Prism software. The result shows that TRPS1 depletion by CRISPR-Cas9 can increase the sensitivity of MCF7 cells to ADR treatment. *H*, MCF7 cells were treated with different concentrations of ADR and the cell viability was examined 2 days after ADR treatment. The IC50 value was calculated using GraphPad Prism software. The relative ADR IC50 assay shows that TRPS1 depletion by siRNAs can increase the sensitivity of MCF7 cells to ADR treatment. *I*, T47D cells were treated with different concentrations of ADR and the cell viability was examined 2 days after ADR treatment. The IC50 value was calculated through the GraphPad Prism software. The relative ADR IC50 assay shows that TRPS1 depletion by siRNAs can increase the sensitivity of T47D cells to ADR treatment. *J*, expression of TRPS1 was diminished in MCF7 cells with siRNAs and the cell viability assay was performed 2 days after treatment with a range of ADR concentrations (1–10 μM). Cell viability assay shows that TRPS1 silenced by siRNAs can decrease the survival rate of MCF7 cells. *K*, MCF7 cells were transduced with TRPS1 siRNAs or control and treated with 5 nM ADR. Representative images of the colony formation assay showed that TRPS1 silencing resulted in a decrease in colony numbers. *L*, Quantification of the invaded colonies in (*K*). Graph bars and error bars represent respectively the mean ± SD of three independent experiments. Statistics analysis was performed using a Student *t* test (two-tailed). ∗*p* < 0.05, ∗∗*p* < 0.01, ∗∗∗*p* < 0.001.

To ascertain an association between TRPS1 expression and chemotherapy sensitivity, we collected and analyzed 109 tumor samples from BC patients undergoing chemotherapy (Table S1). Using immunohistochemistry and chi-squared analysis, we found a strong association between high TRPS1 expression and chemotherapy non-response (Fig. 1*A* and Table 1). Consistent with this, archived data from the public database showed that higher TRPS1 expression was correlated with chemo-nonresponsive BC and worse overall survival in BC patients undergoing chemotherapy (Fig. 1, *B* and *C*). These findings suggest that elevated TRPS1 protects BC cells from chemotherapeutic drugs.

**Table 1:** High TRPS1 expression is associated with resistance in BC patients undergoing chemotherapy

TRPS1 expression	Chemo-sensitive	Chemo-refractory	Total
Low	43	10	53
High	24	32	56
Total	67	42	109

χ^2^ = 16.841 *p* < 0.0001.

Adriamycin (ADR) is a common chemotherapeutic agent used in the treatment of BC treatment (21). To confirm the protective role of TRPS1 in BC cells against chemo-mediated killing, we performed ADR sensitivity assays on a panel of BC cell lines, comparing cells with endogenously high TRPS1 expression (TRPS1^High^) to those with low/undetectable TRPS1 expression (TRPS1^Low^) (Fig. 1*D*). As expected, TRPS1^High^ cells exhibited significantly higher ADR IC50 values than TRPS1^Low^ cells (Fig. 1, *E*–*F* and Table S2). Similarly, we observed that TRPS1^High^ BC cells exhibited significantly higher IC50 values for Etoposide, another chemotherapeutic drug (Table S2). Next, we used the CRISPR-Cas9 system to knock down TRPS1 levels in MCF7 cells (Fig. S1, *A* and *B*); this cell line expresses high levels of TRPS1 and was thus representative of a TRPS1^High^ cell line. TRPS1 knockout MCF7 cells (MCF7^TRPS1-KO^) showed significantly reduced IC50 values upon ADR treatment as compared with the parental cells (MCF7^Par^) (Figs. 1*G* and S1*C*). These MCF7^TRPS1-KO^ cells also showed reduced cell viability and colony number compared to MCF7^Par^ (Fig. S1, *D* and *E*). Similar phenotypic changes were observed in response to ADR treatment in MCF7 and T47D cells depleted for TRPS1 using siRNA (Figs. 1, *H*–*L* and S1, *F*–*I*). Collectively, these findings strongly suggest that elevated TRPS1 expression in BC cells reduces chemosensitivity and contributes to chemoresistance in BC patients.

### Elevated TRPS1 expression promotes DDR hyperactivity in BC cells

**Figure 2:**
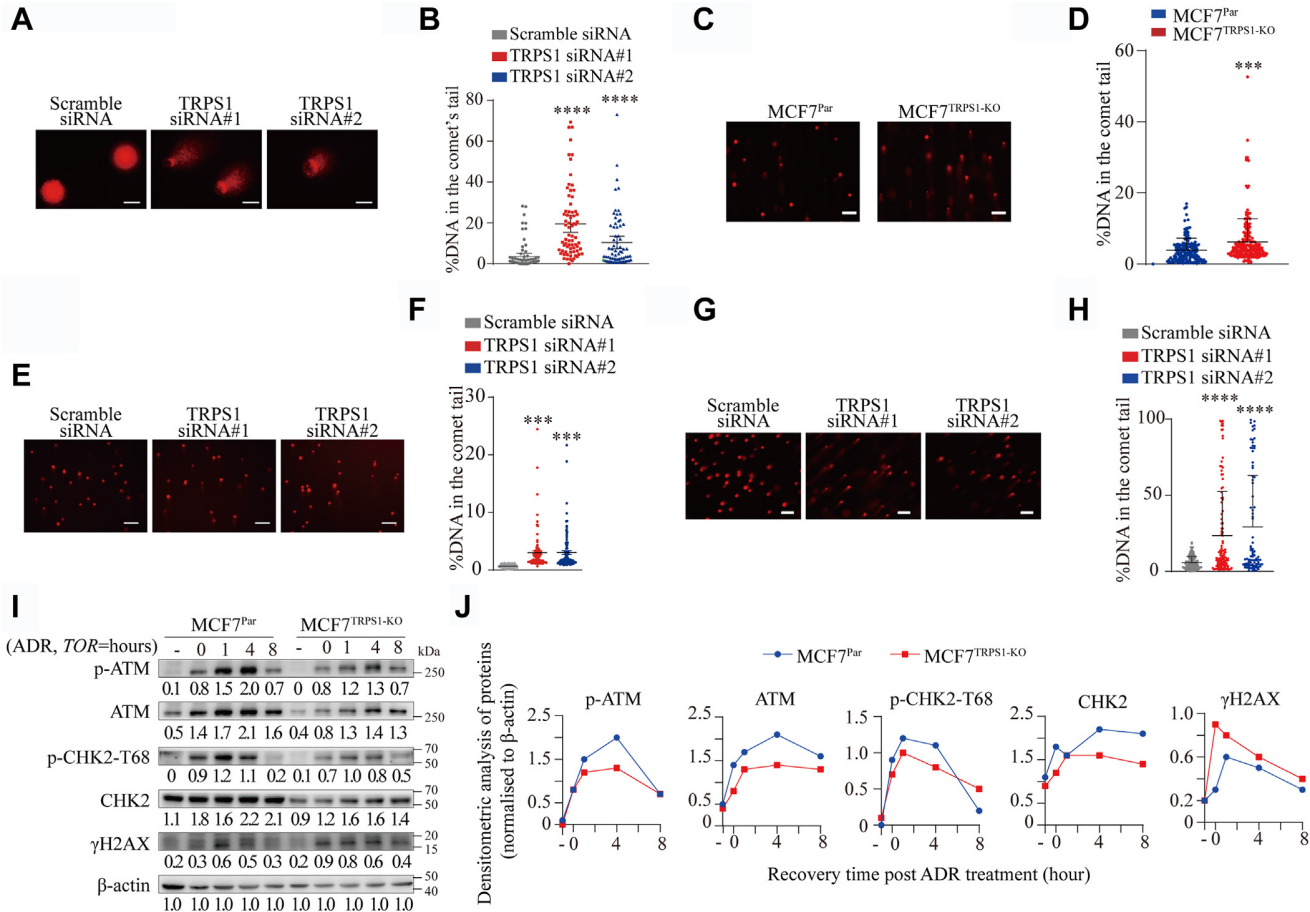
**Reduced TRPS1 leads to DDR defects in BC cells.***A*, representative images of comet assays show that MCF7 cells with TRPS1 silenced by siRNAs have longer comet tails compared to scramble siRNA controls. Scale bar indicates 100 μm. *B*, quantification of DNA percentages in the comet tails shows reduced DNA repair capacity in MCF7 cells with TRPS1 silenced by siRNAs based on the images shown in A. *C*, representative images of comet assays show that ADR-treated MCF7^TRPS1-KO^ exhibits longer comet tails compared to ADR-treated MCF7^Par^. The scale bar indicates 100 μm. *D*, in comet assays, quantification of DNA percentages in the comet tails shows reduced DNA repair capacity in MCF7^TRPS1-KO^ cells compared to MCF7^Par^ cells in C. *E*, representative images of comet assays show that ADR-treated MCF7^Par^ with TRPS1 silenced by siRNAs exhibit longer comet tails compared to scramble siRNA controls. The scale bar indicates 100 μm. *F*, quantification of DNA percentages in the comet tails shows reduced DNA repair capacity in MCF7^Par^ with TRPS1 silenced by siRNAs based on the images shown in E. *G*, Representative images of comet assays show that Etoposide-treated MCF7^Par^ with TRPS1 silenced by siRNAs exhibit longer comet tails compared to scramble siRNA controls. The scale bar indicates 20 μm. *H*, quantification of DNA percentages in the comet tails show reduced DNA repair capacity in MCF7^Par^ with TRPS1 silenced by siRNAs based on the images shown in *G*. *I*, MCF7^Par^ and MCF7^TRPS1-KO^ cells were challenged with 5 μM ADR for 2 h, followed by chase in fresh media as indicated time points. Western blot was performed with the indicated antibodies. Depletion of TRPS1 can negatively affect DNA damage signaling in MCF7 cells, as determined by Western blotting of selected key DDR factors. *J*, densitometric analysis of western blotting in (*I*). Graph bars and error bars represent respectively the mean ± SD of three independent experiments. Statistics analysis was performed using a student *t* test (two-tailed). ∗∗∗*p* < 0.001, ∗∗∗∗*p* < 0.0001.

Most chemotherapeutic drugs, including ADR, kill cancer cells by causing DNA damage (22). To investigate the potential contribution of TRPS1 to the DNA damage response in BC cells, we first performed comet assays in MCF7 cells with TRPS1 knockdown under neutral conditions. We observed that TRPS1 knockdown resulted in MCF7 cells with an increased percentage of DNA in the comet tails (Fig. 2, *A* and *B*). We then performed comet assays in MCF7^TRPS1-KO^ cells after insult with ADR as an inducer of DNA damage. Compared to MCF7^Par^ cells, we found a significantly increased percentage of DNA in the comet tails of MCF7^TRPS1-KO^ cells (Fig. 2, *C* and *D*). Similarly, TRPS1 knockdown by siRNA silencing also resulted in an increased percentage of DNA in the comet tails upon ADR or Etoposide treatment (Fig. 2, *E*–*H*). These results suggest that DNA damage repair is defective in the absence of TRPS1. Ataxia telangiectasia-mutated kinase (ATM) is a master regulator of cellular responses to DSBs, and its phosphorylation (p-ATM) can be used to monitor DNA damage signaling (23, 24). Downstream, we can also measure the DNA damage response through T68 phosphorylation of CHK2, a well-characterized effector of ATM signaling and thus an indicator of ATM activation (25), as well as through phosphorylation of the histone H2A variant, H2AX, which generates γH2AX and serves as a beacon for the presence of DSBs (26). Following ADR treatment, we found that MCF7^TRPS1-KO^ cells exhibited a significantly reduced increase in the levels of ATM protein, p-ATM protein, CHK2 protein, and p-CHK2 protein, as well as a delayed γH2AX upregulation as compared to MCF7^Par^ cells (Fig. 2, *I* and *J*). Moreover, a similar trend was also observed in T47D cells (Fig. S2, *A* and *B*). Together, these results demonstrate that TRPS1 is required for efficient DDR in BC cells.

**Figure 3:**
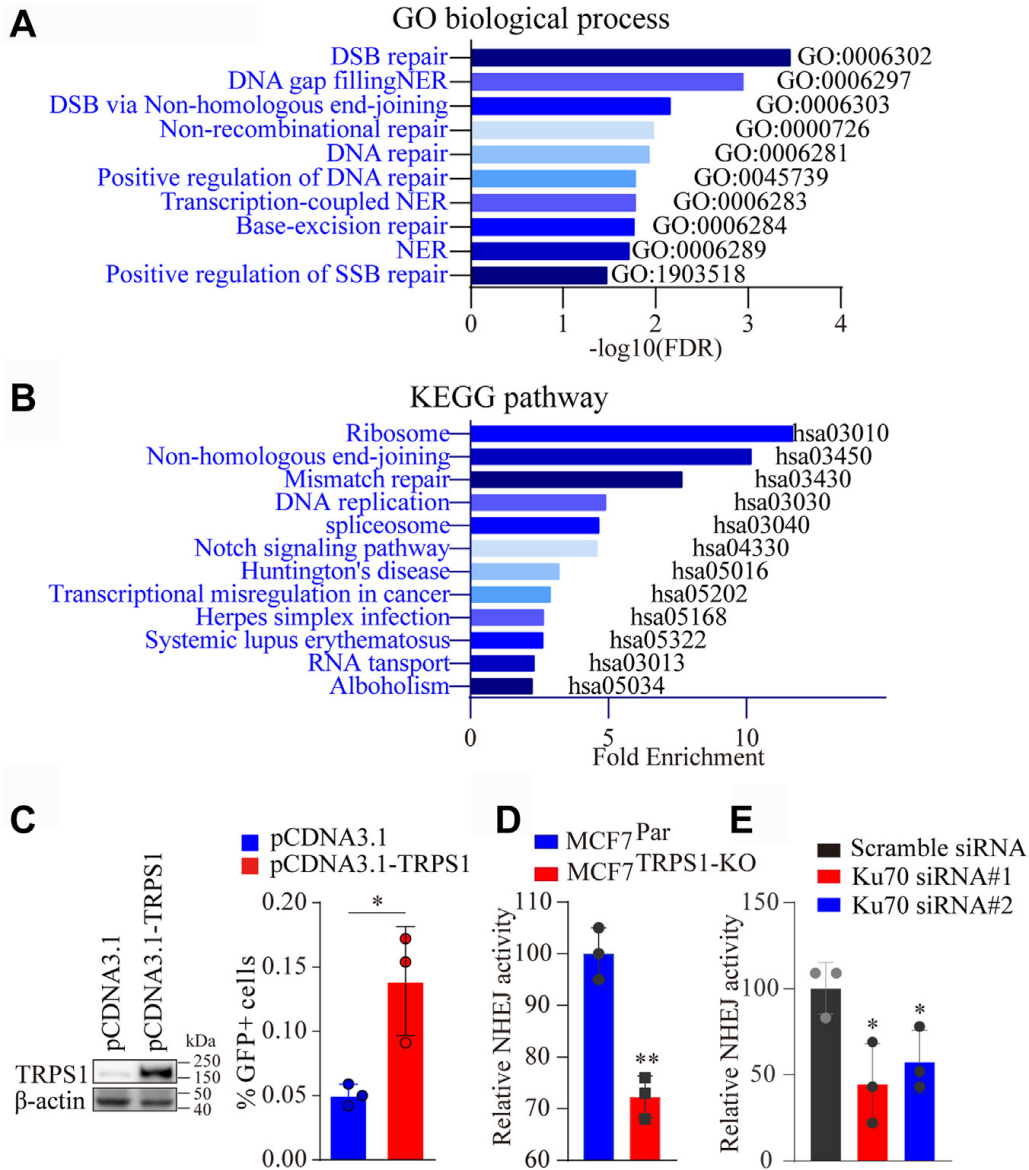
**Elevated TRPS1 expression in BC cells promotes DDR hyperactivity.***A*, TRPS1-binding proteins are enriched in DNA repair-related GO biological processes. *B*, TRPS1-binding proteins are enriched in KEGG pathways, including the NHEJ pathway. *C*, EJ5-GFP-U2OS cells were transfected with pCDNA3.1-TRPS1 or control vector. NHEJ pathway activity was assessed 3 days after the transfection by quantifying %GFP + cells using the flow cytometry analyzer. Overexpression of TRPS1 can significantly enhance NHEJ DNA repair activity. *D*, MCF7^TRPS1-KO^ cells were transfected with pCBASceI and pimEJ5GFP. NHEJ pathway activity was assessed 3 days after the transfection by flow cytometry analysis and quantification of %GFP + cells. TRPS1 knockout results in reduced NHEJ DNA repair activity. *E*, MCF7 cells were transfected with Ku70 siRNAs for 24 h, followed by transfection with pCBASceI and pimEJ5GFP. NHEJ pathway activity was assessed by flow cytometry analysis 3 days after the transfection. Graph bars and error bars represent respectively the mean ± SD of three independent experiments. Statistics analysis was performed using a student *t* test (two-tailed). ∗*p* < 0.05, ∗∗*p* < 0.01, ∗∗∗*p* < 0.001.

Data suggest that hyperactivity of the DDR pathway in cancer cells can prevent such DNA damage-induced cell death (23). Therefore, we rationally hypothesized that elevated TRPS1 expression in BC cells may promote cancer cell insensitivity to DNA-damaging agents by promoting robust DNA repair. To test this hypothesis, we performed gene-ontology (GO) analysis and KEGG pathway enrichment analysis on the proteins co-immunoprecipitated with TRPS1. The GO analysis results show that these proteins are enriched in several DDR-related biological processes, including non-homologous end-joining (NHEJ) for double-stranded DNA breaks (DSBs) (Fig. 3*A* and Dataset S1). DSBs are among the most severe forms of DNA damage and are primarily responsible for most of the cytotoxic effects caused by DNA-damaging agents in patients undergoing chemotherapy (27). DSBs are primarily repaired through homologous recombination (HR) and NHEJ pathways (28, 29), with NHEJ playing a prominent role in humans (30). Notably, the results of the KEGG pathway enrichment analysis show that the NHEJ pathway is the only enriched DDR pathway (Fig. 3*B* and Dataset S1). EJ5SceGFP is a well-established model for testing NHEJ activity in cells, using the percentage of GFP-positive (GFP+) cells as a measure of NHEJ efficiency (Fig. S3, *A* and *B*). Using this model, we show that overexpression and depletion of TRPS1 in cells increase and decrease NHEJ pathway activity, respectively (Fig. 3, *C* and *D*), and we depleted the key NHEJ factor Ku70 in MCF7 cells as a positive control (Figs. 3*E* and S3*C*). These results suggest that TRPS1 enhances DDR activity to antagonize chemo-induced DNA damage in cancer cells.

### TRPS1 is a new DDR protein

We and others have reported that TRPS1 exerts its biological function by acting as a transcription repressor to shape the transcriptional network in normal and diseased cells, including BC cells (13, 15, 31, 32, 33, 34, 35, 36, 37, 38). To explore the mechanism by which TRPS1 promotes DDR, we analyzed the expression of genes known to be involved in DDR based on published RNA-seq data (GSE107023) (17). The analysis showed that the expression of genes known to be involved in DDR was inhibited, not elevated (Fig. S4*A*). In addition, we found no change in the mRNA levels of most of the known DNA repair genes after TRPS1 depletion, regardless of ADR treatment (Fig. S4, *B*–*D*). These results suggest that TRPS1 promotes DDR independent of its transcriptional function.

**Figure 4:**
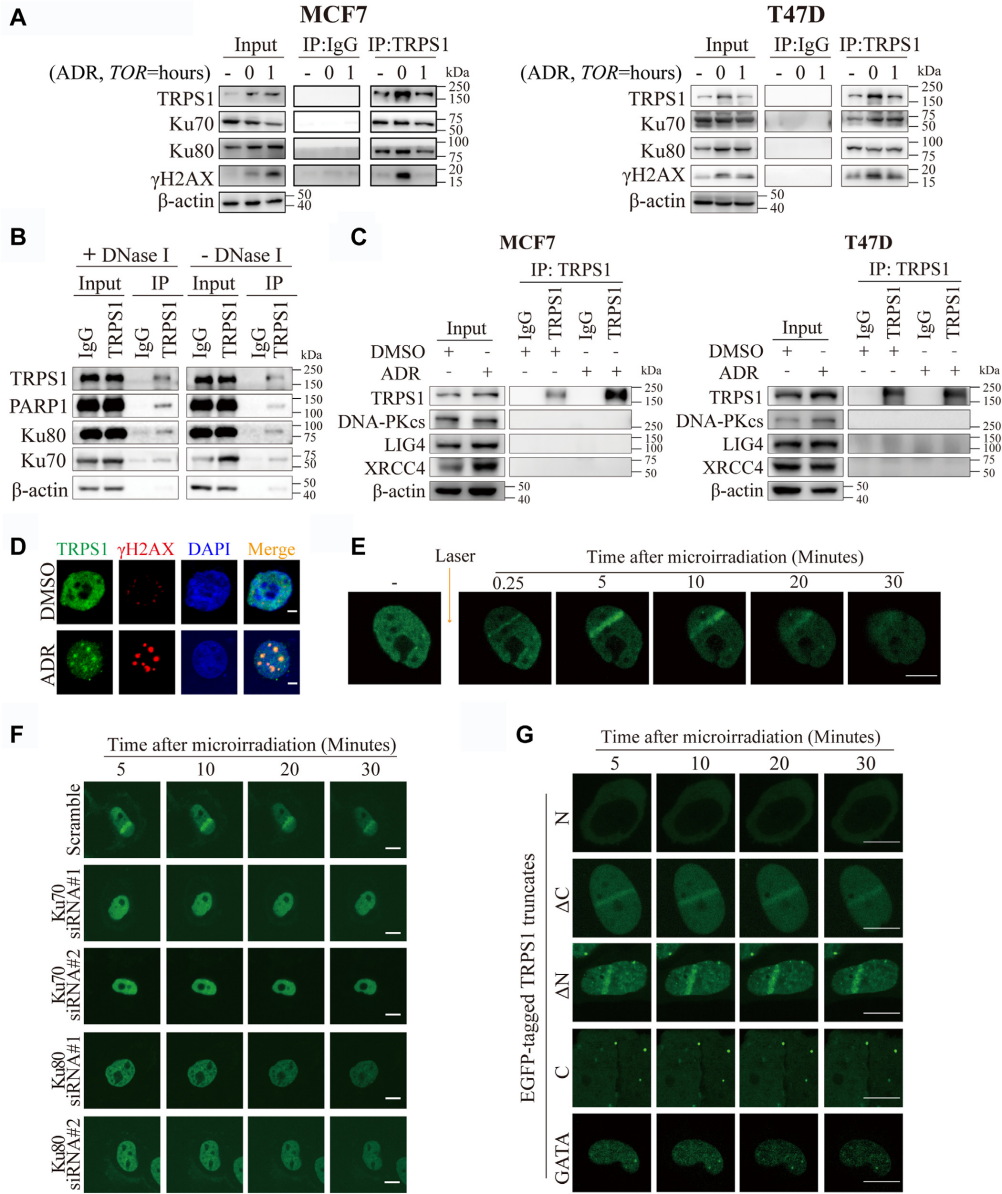
**TRPS1 is a new DDR protein.***A*, MCF7 and T47D cells were challenged with 5 μM ADR for 2 h and then chased in fresh media at the indicated time points. Co-immunoprecipitation (co-IP) was performed with the indicated antibodies and representative co-immunoprecipitation blots show that TRPS1 co-immunoprecipitated with Ku70, Ku80, and γH2AX. *B*, MCF7 cells were collected for lysates and incubated with or without the Deoxyribonuclease I followed by immunoprecipitation with the TRPS1 antibody and control IgG. Representative co-immunoprecipitation blots show the co-immunoprecipitation relationship between TRPS1 and Ku and PARP1, which were resistant to DNase I treatment. *C*, Immunoprecipitation was performed with TRPS1 antibody or control IgG in MCF7 and T47D cells challenged with 5 μM ADR or DMSO, and representative co-immunoprecipitation blots show that the co-immunoprecipitation relationship between TRPS1 and other NHEJ factors (LIG Ⅳ, XRCC4, and DNA-PKcs) were not observed. *D*, representative immunofluorescence staining of γH2AX and TRPS1 in ADR-treated MCF7 cells at 2 h. Immunofluorescence analysis shows that TRPS1 colocalizes with γH2AX. Scale bar: 10 μm. *E*, TRPS1-GFP-transfected U2OS cells were damaged by laser irradiation of the indicated nuclear region. The dynamics of TRPS1-GFP during the DNA damage response on transfected cells were monitored by live cell imaging for 30 min after laser irradiation. Scale bars indicate 25 μm. Representative images of microirradiation assays show dynamic localization of TRPS1 at DNA breaks. *F*, TRPS1-GFP-transfected U2OS cells were damaged by laser irradiation of the indicated nuclear region. The dynamics of TRPS1-GFP during the DNA damage response on control and Ku70 or Ku80 depleted cells were monitored by live cell imaging for 30 min after laser irradiation. Scale bars indicate 25 μm. *G*, U2OS cells were transfected with truncated TRPS1-GFP and damaged by laser irradiation of the indicated nuclear region. The dynamics of truncated TRPS1-GFP during the DNA damage response on transfected cells was monitored by live cell imaging for 30 min after laser irradiation. Representative images show defects in the dynamics of assemblies of different TRPS1 truncation mutants after micro irradiation over time. Scale bars indicate 25 μm.

To investigate whether the promoting effect of TRPS1 on DDR is dependent on non-transcription factor function, TRPS1 protein levels are determined during DDR. We show that TRPS1 protein levels are not altered upon ADR treatment compared to controls and remain relatively stable in cells challenged with ADR after chasing in fresh media at the indicated time points (Fig. S4, *E* and *F*). Furthermore, after insult with ADR, TRPS1 co-immunoprecipitated with several known DRPs, as shown in Fig. 4*A*, including Ku70, Ku80, and γH2AX (Dataset S1). Importantly, the co-immunoprecipitation relationship between TRPS1 and DRPs was not mediated by nucleic acid (Figs. 4*B* and S4*G*). The co-immunoprecipitation relationship between TRPS1 and other NHEJ factors (LIG4, XRCC4 and DNA-PKcs) was not observed (Fig. 4*C*). Using immunofluorescence assays, we show colocalization of TRPS1 with increased expression of γH2AX in ADR-treated MCF7 cells (Fig. 4*D*). Based on these results, we further investigated whether TRPS1 is involved in DDR by directly localizing to DNA breaks in cells. To test this, we performed micro-irradiation assays and found that TRPS1 directly and dynamically localized to the damaged DNA line after laser treatment (Fig. 4*E*). However, we observed that the recruitment of TRPS1 to to the damaged DNA line was significantly abrogated in Ku70-depleted or Ku80-depleted cells (Figs. 4*F* and S4*H*), Taken together, these results suggest that TRPS1 localizes to DNA breaks in cells in a Ku70-and Ku80-dependent manner.

DDR proteins are characterized by their ability to dynamically mobilize to DNA breaks in response to DNA damage (39). However, how this applies to TRPS1 is unclear. By examining the domain organization, we found that TRPS1 contains one GATA domain flanked by an NTD and a CTD (Fig. S4*I*). To ascertain which of these domains is required for TRPS1 recruitment to DNA break sites, we further characterized the role of these domains in the context of DDR using micro-irradiation assays. We found that, whereas the truncated forms of TRPS1 retaining the GATA domain localized to DNA break sites, whereas the GATA domain alone failed to be recruited, indicating that the GATA domain is required but not sufficient for TRPS1 recruitment to DNA break sites (Fig. 4*G*). Thus, these results suggest that TRPS1 enhances DDR by functioning as a new DDR protein rather than a transcriptional regulator and that the GATA domain and Ku are essential for TRPS1 function in this process.

### PARylation regulates TRPS1 redistribution to DNA breaks in the DDR

**Figure 5:**
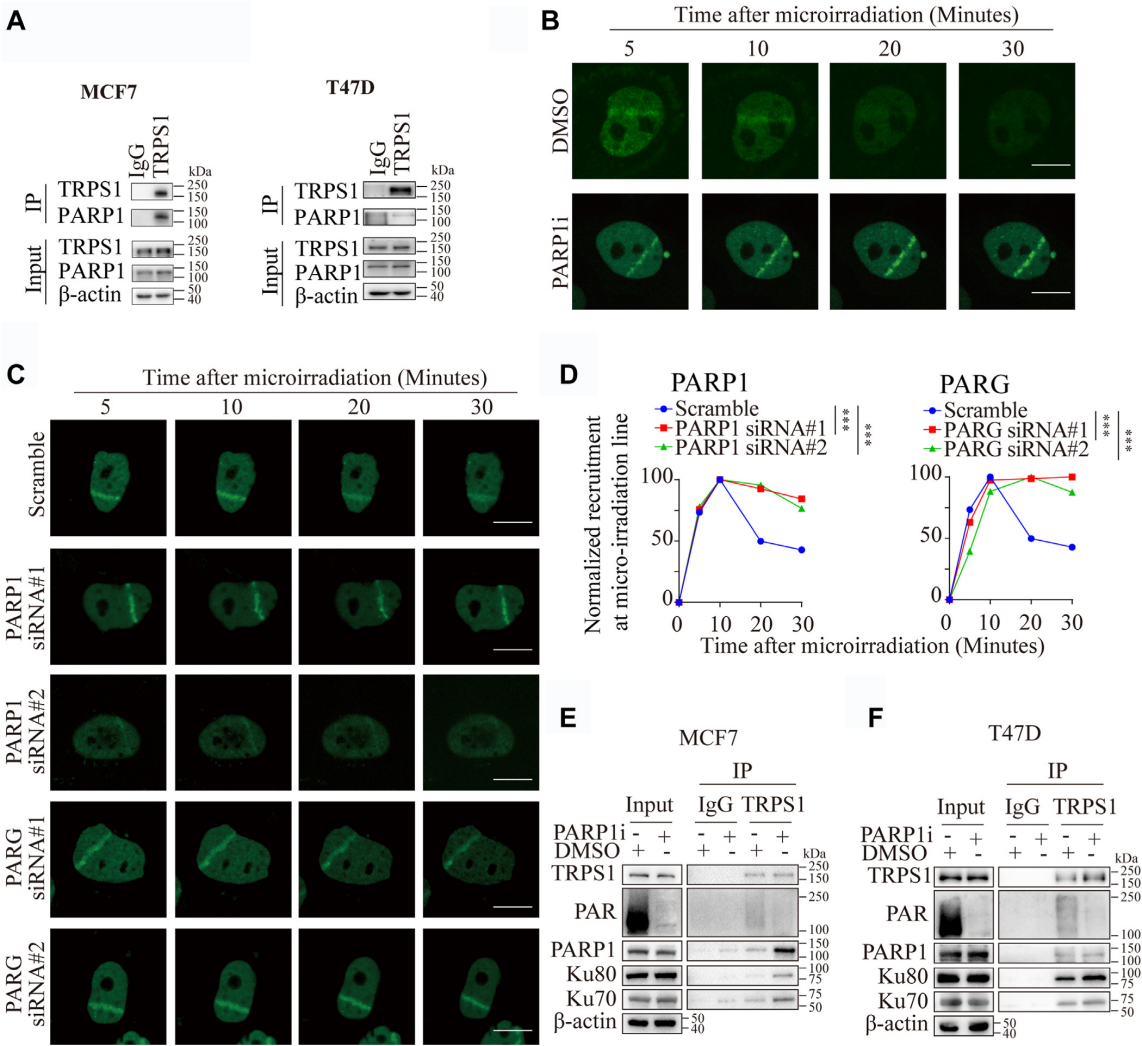
**TRPS1 co-immunoprecipitated with PARP1.***A*, MCF7 and T47D cells were collected for lysates and Co-IP with the TRPS1 antibody or control IgG. Representative Co-IP blots show TRPS1 co-immunoprecipitated with PARP1 in BC cells. *B*, TRPS1-GFP-transfected U2OS cells were damaged by laser irradiation of the indicated nuclear region. The dynamics of TRPS1-GFP during the DNA damage response to inhibition of PARP1 (PARP1i) by AG14361 (1 μM) was monitored by live cell imaging for 30 min after laser irradiation. Inhibition of PARP1abrogates the reversible property of TRPS1 enriched in damaged DNA compared to the control treatment (DMSO). Scale bars indicate 25 μm. *C*, TRPS1-GFP-transfected U2OS cells were damaged by laser irradiation of the indicated nuclear region. The dynamics of TRPS1-GFP during the DNA damage response on control and PARP1 or PARG-depleted cells was monitored by live cell imaging for 30 min after laser irradiation. PARP1 or PARG depletion similarly abolishes the reversibility of TRPS1 in forming reversible compartments enriched at sites of DNA damage. Scale bars indicate 25 μm. *D*, quantification of the images in (*C*) shows the normalized recruitment of TRPS1 at DNA break lines generated by microirradiation. *E*, MCF7 cells were treated with AG14361 for 24 h, then lysates were collected and immunoprecipitated with the TRPS1 antibody or control IgG. Co-IP shows that the co-immunoprecipitation relationship between TRPS1 and DRPs is unaffected by PARylation inhibition. *F*, T47D cells were treated with AG14361 for 24 h, then lysates were collected and immunoprecipitated with the TRPS1 antibody or control IgG. Co-IP shows that the co-immunoprecipitation relationship between TRPS1 and DRPs is unaffected by PARylation inhibition. Graph bars and error bars represent respectively the mean ± SD of three independent experiments. Statistics analysis was performed using a student *t* test (two-tailed). ∗∗∗*p* < 0.001.

Post-translational modifications (PTMs) on DRPs regulate their redistribution to DNA breaks during DDR, which is considered an essential event for efficient DDR (39). Of particular interest is PARylation, which is considered to be one of the most important reversible PTMs in DDR to dynamically localize DRPs to at sites of DNA damage (40). Poly [ADP-ribose] polymerase 1 (PARP1) is a major PARylation enzyme in the DDR, accounting for 80% to 90% of DNA damage-induced PARylation events. PARP1 also facilitates the synthesis of PAR chains, which provide a platform for the recruitment of DDR proteins (41). We identified and confirmed PARP1 as another known DDR protein that co-immunoprecipitates with TRPS1 (Dataset S1 and Fig. 5*A*), with evidence that the GATA domain is required for the co-immunoprecipitation (Fig. S5*A*). In addition, we demonstrated a link between TRPS1 and PARylation modifications using immunoprecipitation assays (Fig. S5, *B*–*D*). We next sought to determine whether and how PARylation affects the redistribution of TRPS1 to DNA breaks in the DDR. Using microirradiation assays, we found that inhibition of PARP1 by AG14361 abolished the disassembly but not the assembly of TRPS1 at DNA damage lines (Fig. 5*B*). PARylation in the DDR is a reversible process that occurs primarily through the activity of PAR glycohydrolase (PARG), a major dePARylation enzyme (42). Similar to PARP1 silencing, we found that PARG silencing abolished the disassembly but not the assembly of TRPS1 at DNA-damaged sites (Figs. 5, *C*–*D* and S5, *E*–*F*). In addition, immunoprecipitation assays revealed that the co-immunoprecipitation relationship between TRPS1 and DRPs, including Ku70, Ku80 and PARP1, was not altered by PARylation inhibition (Fig. 5, *E* and *F*), suggesting that PARylation is required for the release of TRPS1 from DNA-damaged sites. Taken together, these results suggest that PARylation is required for the redistribution of TRPS1 to DNA breaks in the DDR.

### TRPS1 PARylation is required for its redistribution to DNA breaks

**Figure 6:**
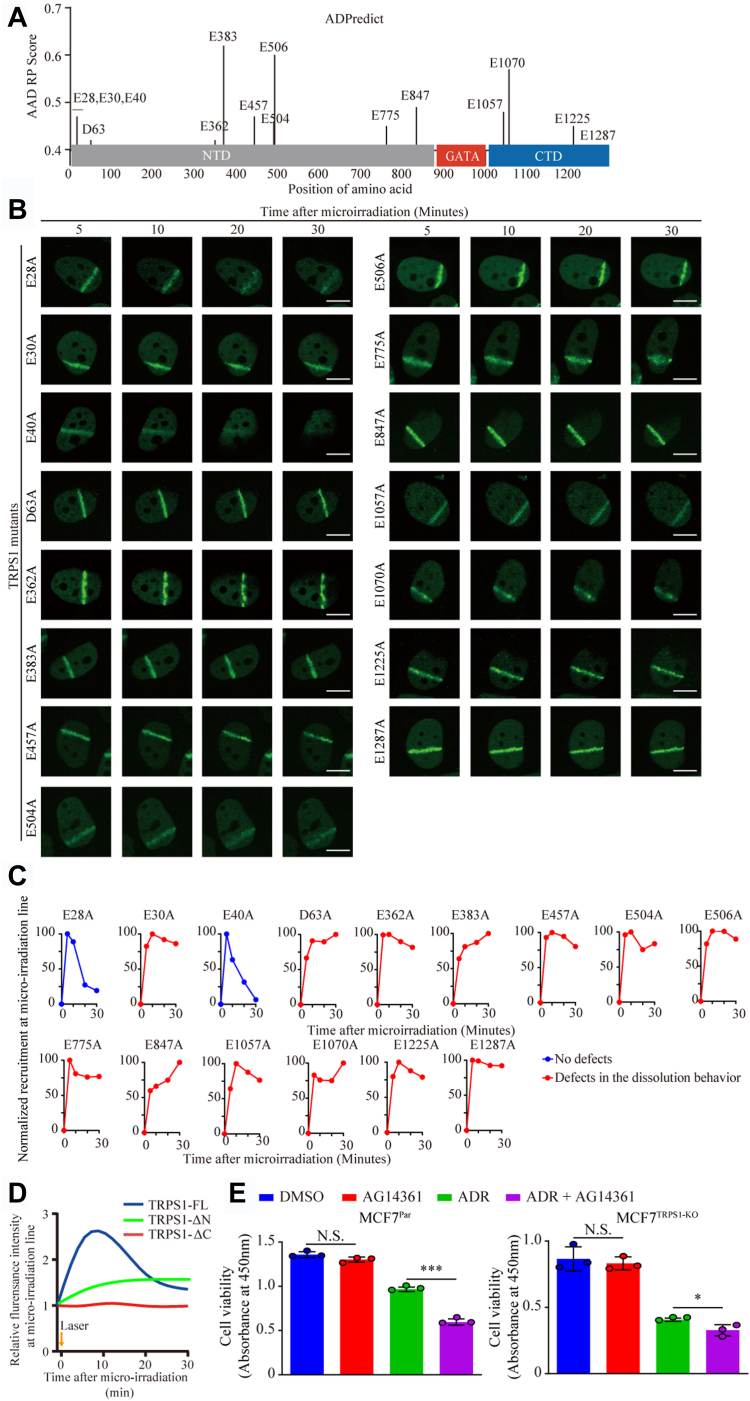
**PARylation regulates the dynamics of TRPS1 assembly at DNA breaks in the DDR.***A*, using the ADPredict server, we identified 15 PARylation sites in the NTD and CTD of TRPS1 but not in the GATA domain, with high confidence (AAD RP score > 0.4). *B*, U2OS cells were transfected with mutant TRPS1-GFP and damaged by laser irradiation of the indicated nuclear region. The dynamics of mutant TRPS1-GFP during the DNA damage response on transfected cells was monitored by live cell imaging for 30 min after laser irradiation. Representative images taken from microirradiation assays show the assembly behavior of TRPS1-carrying mutations at each of the predicted PARylation sites. Scale bars indicate 25 μm. *C*, Graphs showing the normalized recruitment of mutated TRPS1 PARylation sites to DNA break lines generated by microirradiation. *D*, U2OS cells were transfected with EGFP-tagged TRPS1-△C and TRPS1-△N and damaged by laser irradiation of the indicated nuclear region. Assembly of EGFP-tagged TRPS1-△C and TRPS1-△N to DNA break lines during the DNA damage response on transfected cells was monitored by live cell imaging for 30 min after laser irradiation. Quantification of relative assembly of EGFP-tagged TRPS1-△C and TRPS1-△N at microirradiation lines compared to EGFP-tagged full-length TRPS1 over incubation times for Figs. 4*C* and S4*G*. *E*, MCF7^Par^ and MCF7^TRPS1-KO^ cells were treated with AG14361 and ADR for 48 h. Then, cell viability was measured and shows that PARP1 inhibition by AG14361 efficiently sensitizes MCF7^TRPS1-KO^ cells to ADR treatment compared to parental MCF7 (MCF7^Par^) cells. Graph bars and error bars represent respectively the mean ± SD of three independent experiments. Statistics analysis was performed using one-way ANOVA. ∗*p* < 0.05, ∗∗∗*p* < 0.001, and N.S., not significant.

To determine whether these PARylation redistribution effects are conferred by direct PARylation on TRPS1, we used the ADPredict server to predict potential PARylation sites (43). The server predicted 15 PARylation sites in TRPS1 (AAD RP score > 0.4) located within the CTD and NTD (Fig. 6*A*). We tested these sites using microirradiation assays, generating TRPS1 mutants with a single D/E to A mutation at each site. We found that TRPS1 recruitment to DNA break sites was unaffected, but that 13 of the 15 TRPS1 mutants exhibited defective dissolution behaviors reminiscent of those behaviors observed upon abolition of PARylation and dePARylation (Fig. 6, *B* and *C*). Furthermore, immunoprecipitation assays showed that the co-immunoprecipitation relationship between TRPS1 and proteins involved in DNA repair was still present in these mutations (Fig. S6*A*). Since the predicted 15 PARylation sites in TRPS1 are located within the CTD and NTD, and we tested and confirmed the defective dissolution behavior of truncated TRPS1 (truncated CTD or NTD) (Figure 4, Figure 6*D* and 4, *C* and *G*). Thus, these results suggest that the redistribution of TRPS1 is regulated by PARylation.

Importantly, we determined the ADR sensitivity of TRPS1 mutations on MCF7^TRPS1-KO^ cells and found that there was increased sensitivity to ADR in MCF7^TRPS1-KO^ cells with TRPS1 mutations compared to MCF7^TRPS1-KO^ cells with full-length TRPS1 (Fig. S6, *B* and *C*). Finally, we sought to confirm whether inhibition of PARylation with the PARP inhibitor, AG14361, could increase the sensitivity of MCF7^Par^ cells to ADR. As expected, we found that MCF7^Par^ cells were more sensitive to ADR in the presence of AG14361, with a reduced sensitizing effect observed in MCF7^TRPS1-KO^ (Fig. 6*E*). Taken together, we demonstrate that TRPS1 is a new DDR protein that cooperates with reversible PARylation modifications to facilitate DDR for chemoresistance in BC patients.

## Discussion

DNA-damaging agents are frequently administered to patients with BC but with limited success. This lack of response has led to an urgent unmet need to elucidate the mechanisms driving resistance to these chemotherapeutic regimens (2, 44). In the present study, we demonstrate that elevated TRPS1 expression in BC cells is a causal factor in protecting cancer cells from the cytotoxicity of chemotherapy. This not only positions TRPS1 as a new biomarker for predicting chemotherapy response in BC patients but also as a new druggable target for the development of chemo-sensitizers to improve the clinical efficacy of chemotherapy. Furthermore, we characterize TRPS1 as a novel DRR protein that cooperates with reversible PARylation for DDR, thus providing new insights into this refractory mechanism.

DDR is a dynamic, multistep process that is tightly controlled by the spatiotemporal assembly of DRPs, which form transient DNA repair compartments for the efficient repair of damaged DNA in cells (39). PARylation is an important type of PTM in the DDR that occurs at early stages of repair where PAR is abundant (45). PAR has documented roles in regulating chromatin organization and as a platform for DRP recruitment at damaged DNA sites (39). Interestingly, we show that abrogation of PARylation results in defective disassembly for TRPS1 at DNA lesions without arresting recruitment. However, inhibition of PARP1 with the inhibitor AG14361 reduces the PARs that co-immunoprecipitate with TRPS1. TRPS1 is an atypical GATA transcription factor that acts as the only transcriptional repressor in the GATA family (12). In our current study, we demonstrated that TRPS1 regulates DDR in a manner independent of its transcription factor function. Notably, zinc-finger proteins can interact with DNA and PAR (46): TRPS1 contains three distinct zinc-finger domains and TRPS1 inhibition can cause BC cells to accumulate in S phase (47). We show that TRPS1 depletion reduces the sensitivity of BC cells to ADR when PARylation is also inhibited. These results suggest that TRPS1 may not only function in the early stages of DDR but may also be required for PARP1 function in DDR.

Several PARP inhibitors, including Olaparib, Rucaparib, Niraparib, and Talazoparib, have been approved by the US Food and Drug Administration for the treatment of ovarian cancer and BC (48). In addition to advocating TRPS1 as a new target for the development of chemosensitizers for BC, our data provide evidence for the beneficial effect of clinically approved PARP inhibitor drugs in the treatment of chemo-refractory BC with elevated TRPS1, thus providing a ready-to-use pharmacological approach to overcome chemo-failure in patients with BC. The pharmacodynamic properties of PARP inhibitors have been documented to exploit synthetic lethality, including their applications in the treatment of BC with *BRCA* mutations (49, 50). In contrast, we propose that BC cells with elevated TRPS1 expression can be sensitized to chemotherapeutic drugs using a PARP inhibitor *via* altered TRPS1 disassembly behaviors. Standing on TRPS1, our data support a conceptually new mechanism behind the pharmacodynamic properties of PARP inhibitors that goes beyond exploiting synthetic lethality, where PARP inhibitors interfere with the assembly microenvironment of DNA repair compartments in the DDR, independent of their targets. Thus, PARP inhibitors could be considered as the first clinically approved drug to exploit the essential role of the proper disassembly behavior of DRPs in disease states, and thus reinforcing a new paradigm for the innovation of therapeutic strategies *via* targeting the assembly/disassembly behavior of DRPs.

## Experimental procedures

### Cell culture and transfection

All cell lines (ATCC) were cultured in medium (WISENT) and supplemented with 10% fetal bovine serum (WISENT) and 1% penicillin/streptomycin (WISENT). Cells were incubated at 37 °C in humidified air containing 5% CO_2_. Vector transfections were performed using Lipofectamine 3000 (Thermo Scientific), and siRNAs transfections were performed using RNAiMAX (Thermo Scientific) in serum-free Opti-MEM medium (Thermo Scientific) according to the manufacturer’s instructions. Sequences for siRNAs are listed in Table S3.

### Generation of cDNA constructs

PEGFP-N2-TRPS1 and truncations were amplified by PCR from pcDNA3.1-FLAG-TRPS1 using the primers listed in Table S4 with BamHI and EcoRI (New England Biolabs) restriction sites. Single D/E to A mutation of EGFP-tagged TRPS1 was generated by PCR-based, site-directed mutagenesis using Mut Express MultiS Fast Mutagenesis Kit (Vazyme C215–01), according to the manufacturer’s instructions. Primer sequences are listed in Table S5. All plasmids were verified by sequencing (GENEWIZ).

### Co-IP

For Flag-tagged co-IP, cell lysates were incubated with 15 μl Anti-FLAG M2 Protein G Magnetic Beads (Sigma) at 4 °C overnight. Protein G beads (Thermo Scientific) conjugated with anti-TRPS1 or anti-PAR antibodies were used for TRPS1 or PAR co-IP, respectively. Following precipitation, the beads were then repeatedly centrifuged and washed in 50 mM Tris-HCl, pH 7.5, 150 mM NaCl, 1% Triton X-100, 1 mM EDTA. Total proteins and IP complexes were separated by SDS-PAGE with the indicated primary and corresponding secondary antibodies. Each co-IP experiment was performed at least three times in independent experiments.

### Chemosensitivity assays

#### Cell viability assay

Cell viability was analyzed using the CCK-8 kit (Dojindo Laboratories), according to the manufacturer’s instructions.

#### Clonal formation assay

Cells were seeded into 6-well plates at a density of 500 cells per well. After 24 h, cells were treated with Adriamycin (ADR) (Selleck, S1208) for 14 days. At the end of treatment, the colonies were fixed in 4% paraformaldehyde (PFA) and stained with Giemsa, according to standard laboratory protocols.

#### IC50 assay

Cells were cultured in 96-well plates and were dosed with the indicated compounds. IC50 values were calculated by fitting a dose–response curve to normalized data using GraphPad Prism software.

### Clinical samples

Breast tumor tissues from patients with chemotherapy treatment were obtained from the Nanjing Maternity and Child Health Care Hospital. The collection of clinical samples was carried out in accordance with the approved guidelines of the Nanjing Maternity and Child Health Care Hospital.

### Immunohistochemical staining

Primary breast tumor sample sections on slides were fixed with 4% PFA, embedded, de-waxed and rehydrated following standard procedures. Antigen retrieval was then performed by heating and incubating the slides in 1% hydrogen peroxidase. Subsequently, the slides were incubated with the indicated antibodies. Staining was performed using the HRP-IHC kit (Abcam, ab64261), according to the manufacturer’s instructions.

### DNA repair assays

#### Comet assay

Cell suspensions were lysed in a buffer containing 2.5 M NaCl, 100 mM Na_2_EDTA, 10 mM Tris-HCl, 1% Triton X-100, 10% DMSO (pH 10). DNA was denatured under alkaline conditions (1 mM Na_2_EDTA, 300 mM NaOH, pH > 13), and electrophoresed under 15 V for 12 min in cold buffer to resolve fragmented DNA from intact DNA. Gels were stained with GelRed Nucleic Acid Gel Stain (Biotium, 41,003) and visualized by fluorescence microscopy under a 10 × objective. DNA samples appeared as a comet in shape, with comet parameters analyzed using Comet Assay software (http://casplab.com/download).

#### NHEJ repair assay

NHEJ repair assay was performed as described by Bennardo and colleagues (51). EJ5-GFP-U2OS cells (1 × 10^5^ per well), grown in 6-well plates, were co-transfected with the I-SceI expression plasmid and either pCDNA3.1 or pCDNA3.1-TRPS1. At 48 h after transfection, all cells were harvested and analyzed for the presence of GFP-positive cells by flow cytometry. NHEJ repair was reported as the ratio of GFP-positive cells as compared with the total number of cells. This ratio normalized the repair events to the transfected controls.

#### Laser microirradiation assay

Laser microirradiation was carried out following procedures described previously (52). HeLa cells with overexpression of either EGFP-tagged TRPS1 or its truncation mutants were grown on thin glass-bottom plates and irradiated with an ultraviolet laser (16 Hz pulse, 41% laser output). Images were taken using a Nikon A1 confocal imaging system.

### Real-time quantitative PCR

Total RNA was extracted and purified from breast cancer cell lysates *via* using FastPure Cell/Tissue Total RNA Isolation Kit (RC101, Vazyme) and cDNA was synthesized by using HiScript II Q RT SuperMix for qPCR (R222–01, Vazyme). Quantitative PCR was performed using ChamQ SYBR qPCR Master Mix (High ROX Premixed) (R341–02, Vazyme) in a StepOnePlus Real-time PCR system (Thermo Fisher Scientific). The primer sequences are available in Table S6.

### Western blotting

Cells were lysed in RIPA buffer supplemented with PMSF on ice. Proteins were separated by SDS-PAGE and transferred onto PVDF membranes (Millipore) as per standard laboratory protocols, and then blotted with specific antibodies as outlined in Table S7.

### Statistics

Experiments were repeated at least three times. Statistics analyses were performed using a student *t* test (two-tailed) or one-way ANOVA with GraphPad Prism seven. All results are presented as mean ± SD unless otherwise stated. (∗*p* < 0.05; ∗∗*p* < 0.01; ∗∗∗*p* < 0.001; ∗∗∗∗*p* < 0.0001)

## Data availability

All raw data generated in this study are available upon request from the corresponding author. The data analyzed in this study were obtained from GEO at GSE20194, GSE22093, GSE23988, GSE25055, GSE25065, and GSE42822.

## Supporting information

This article contains supporting information.

## Conflict of interests

The authors declare that they have no conflicts of interest with the contents of this article.
