# Supplementary material for: GATA factor TRPS1, a new DNA repair protein, cooperates with reversible PARylation to promote chemoresistance in patients with breast cancer
Source: J Biol Chem. 2024 Sep 12;300(10):107780. doi: 10.1016/j.jbc.2024.107780 (PMC11490888; doi:10.1016/j.jbc.2024.107780)
Supplement: Supporting information [file mmc1.docx]

**Supporting Material**

**GATA factor TRPS1, a new DNA repair protein, cooperates with reversible PARylation to promote chemoresistance in breast cancer patients**

Jun Zhang^1,7^, Yatao Chen^1,7^, Xue Gong^1,2,7^, Yongfeng Yang^3,7^, Yun Gu^2,7^, Ling Huang^1,7^, Jianfeng Fu^3^, Menglu Zhao^1^, Yehong Huang^1^, Lulu Li^1^, Wenzhuo Liu^1^, Yajie Wan^1^, Xilin He^1^, Zhifang Ma^1,2^, Weiyong Zhao^4^, Meng Zhang^1^, Tao Tang^1^, Yuzhi Wang^1^, Jean Paul Thiery^5,*^, Xiaofeng Zheng^3,*^, Liming Chen^1,6,8,*^

^1^Department of Biochemistry, School of Life Sciences, Nanjing Normal University, Nanjing, China

^2^Women's Hospital of Nanjing Medical University, Nanjing Women and Children’s Healthcare Hospital, Nanjing, China

^3^State Key Lab of Protein and Plant Gene Research, Department of Biochemistry and Molecular Biology, School of Life Sciences, Peking University, Beijing, China

^4^Department of Radiation Oncology, Affiliated Hopital of Integrated Traditional Chinese and Western Medicine,Nanjing University of Chinese Medicine, Nanjing, China

^5^Institute of Molecular and Cell Biology, A*STAR, Singapore 138673

^6^Jiangsu Institute of Cancer Research, Jiangsu Cancer Hospital, the Affiliated Cancer Hospital of Nanjing Medical University, Nanjing, China

^7^These authors contributed equally

^8^Lead Contact

^*^Correspondence:

chenliming1981@njnu.edu.cn; tjp@visitor.nus.edu.sg; xiaofengz@pku.edu.cn

**Supporting Information Figures**

**
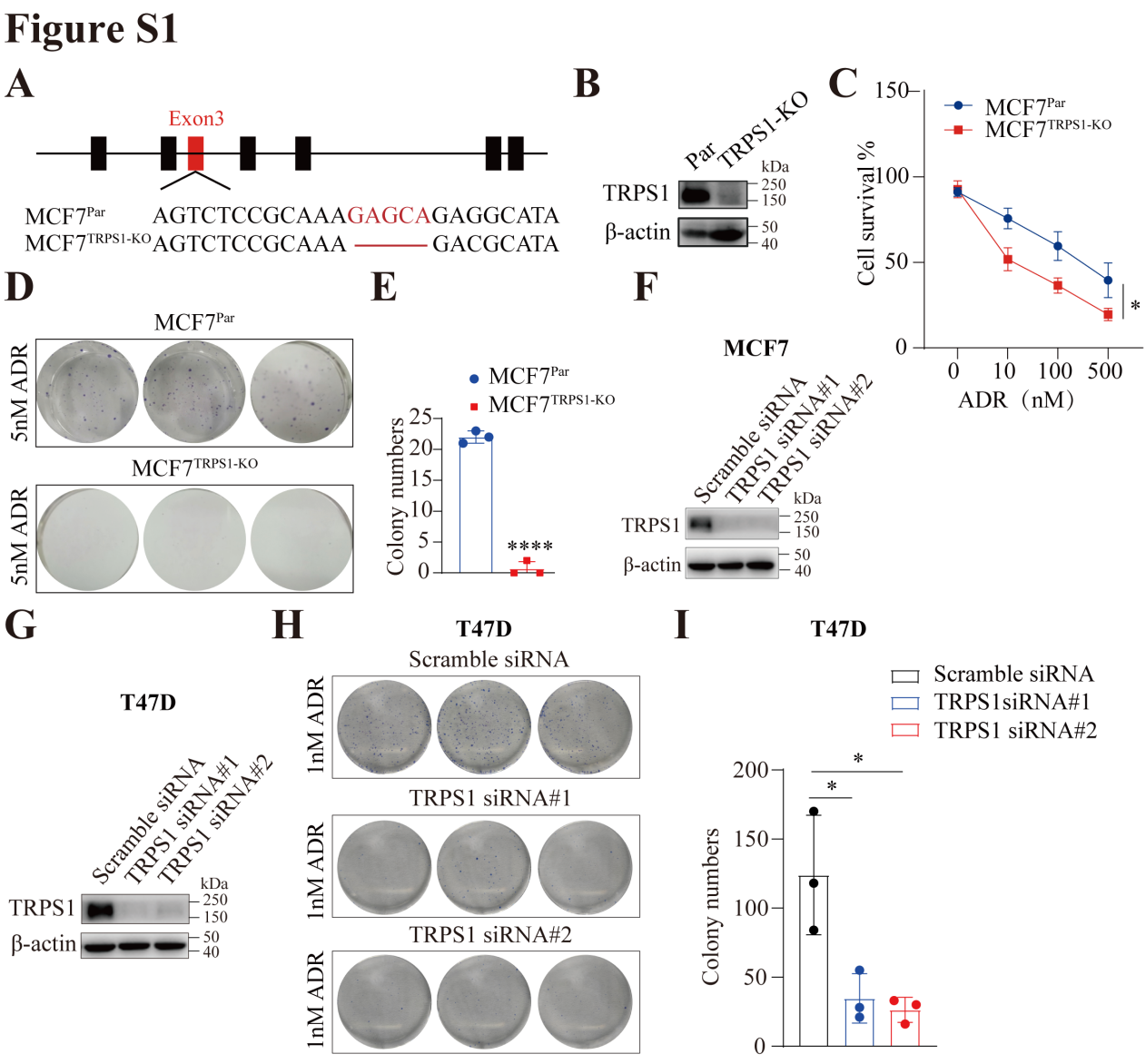
**

**
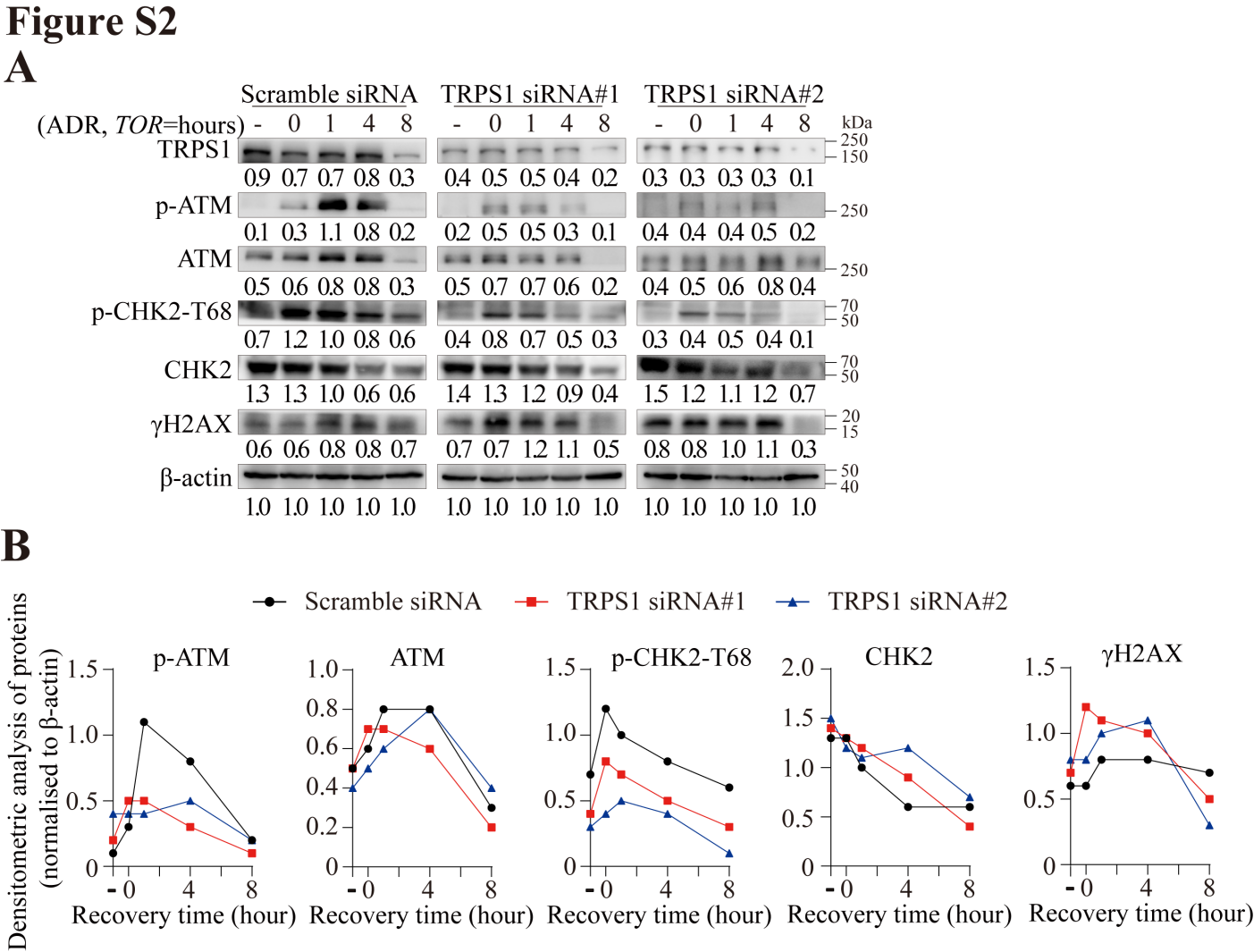
**

**
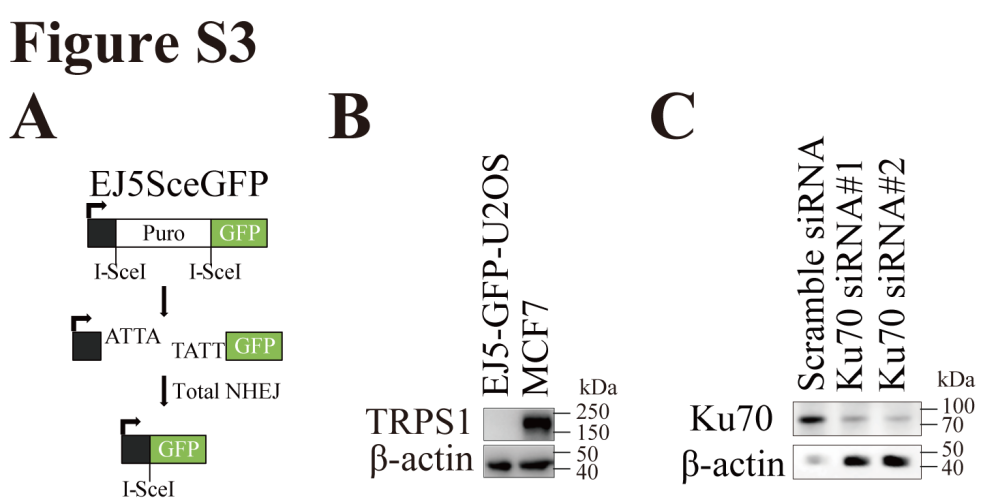
**

**
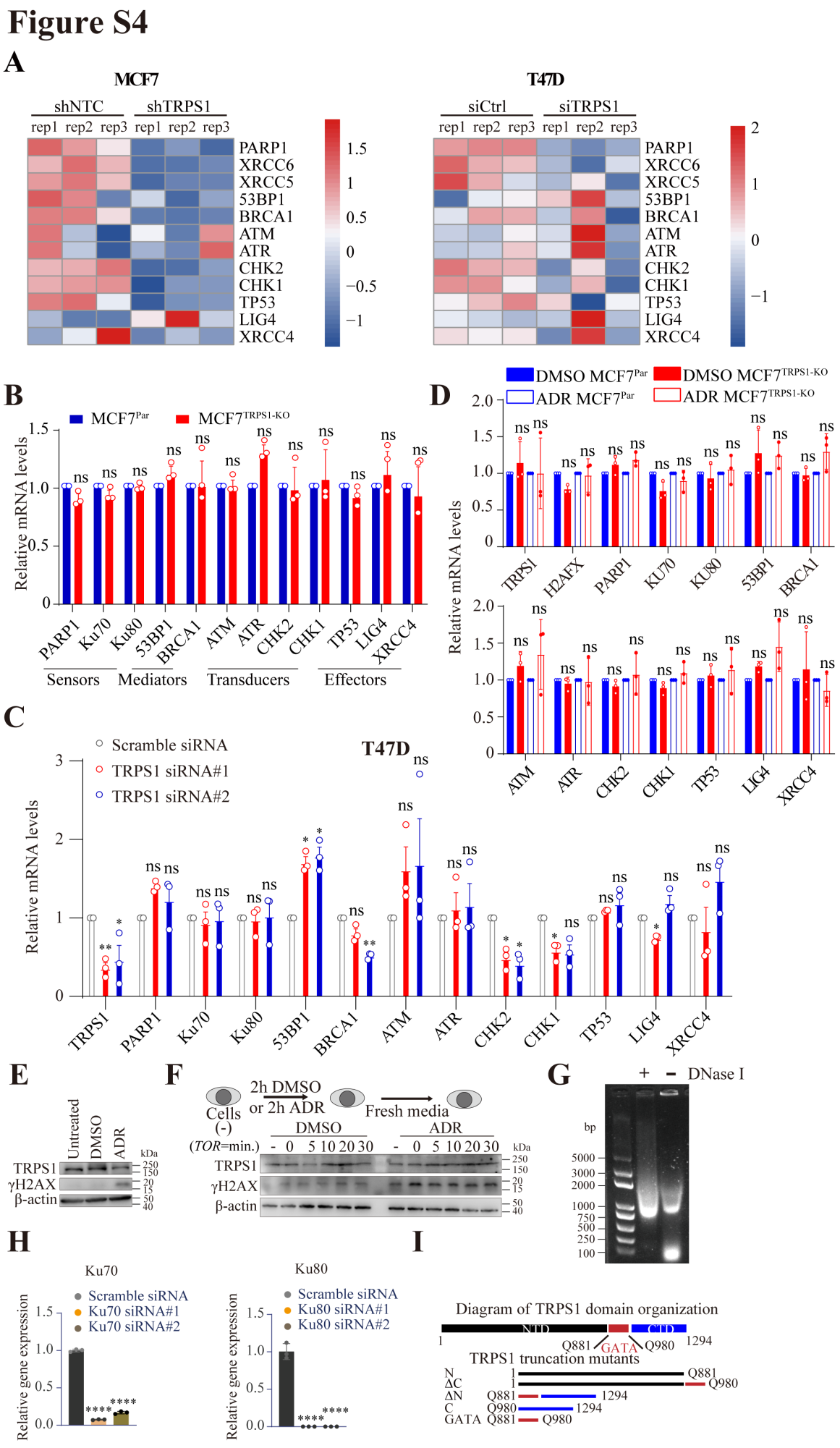
**

**
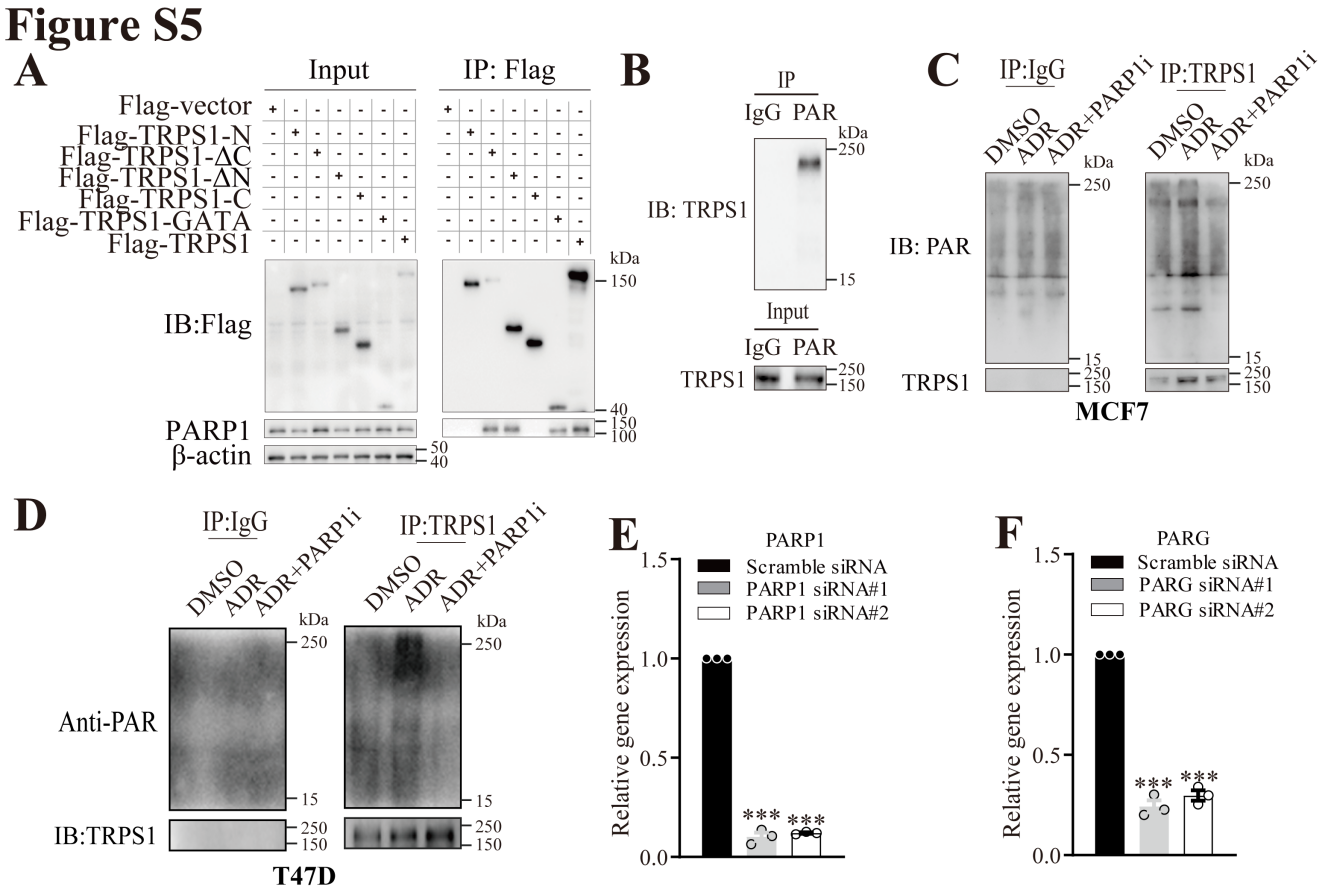
**

**
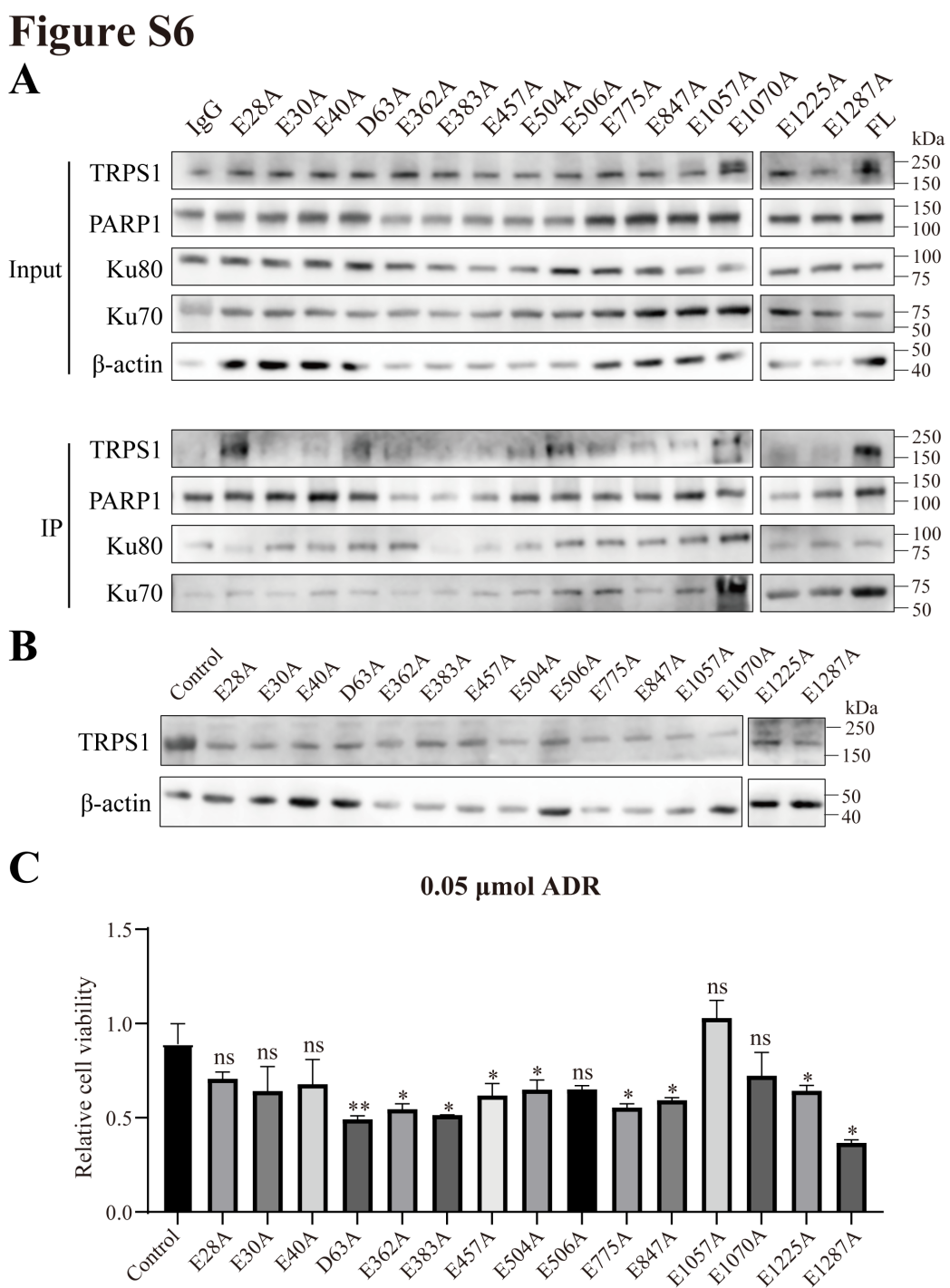
**

**Supporting Information Figure legends**

**Figure S1.TRPS1 depletion enhances BC cell chemosensitivity.**

**(A)** The sequencing result of TRPS1 gene in MCF7^TRPS1-KO^ and MCF7^Par^ shows that five nucleotides highlighted in red color of the protein coding region in *TRPS1* gene is successfully deleted by CRIPR-Cas9 in MCF7^TRPS1-KO^. **(B)** A representative western blot shows that TRPS1 is successfully depleted by CRIPR-Cas9 in MCF7 cells. **(C)** MCF7^Par^ and MCF7^TRPS1-KO^ cells were treated with a range of ADR concentrations (10 to 500 nM) for 48 hours and cell survival assay was performed. The cell survival shows that MCF7^TRPS1-KO^ cells can decrease the survival rates of cells as compared with MCF7^Par^ cells. **(D)** Representative images of colony formation assay show that MCF7^TRPS1-KO^ cells exhibit reduced colony numbers compared to MCF7^Par^ cells upon 5nM ADR treatment. **(E)** Quantification of the validated colonies in (D). **(F)** A representative western blot shows that TRPS1 is successfully silenced using siRNAs in MCF7 cells. **(G)** A representative western blot shows that TRPS1 is successfully silenced using siRNAs in T47D cells. **(H)** Representative images of colony formation assay show that T47D cells with TRPS1 silenced by siRNAs exhibit reduced colony numbers upon 1nM ADR treatment. **(I)** Quantification of the validated colonies in (H). Graph bars and error bars represent respectively the mean±SD of three independent experiments. Statistics analysis was performed using a student t-test (two-tailed) or one-way ANOVA. *P<0.05, ****P<0.0001.

**Figure S2. Reduced TRPS1 led to DDR defect in BC cells.**

**(A)** T47D cells were tansfected with TRPS1 siRNAs or control for 48 hours, subsequently challenged with 2.5 μM ADR for 2 hours, then chased in fresh media as indicated time points. Western blot was performed with antibodies indicated. Depletion of TRPS1 can negatively affect DNA damage signaling in T47D cells, determining by western blotting of selected important DDR factors. Inhibition of TRPS1 can negatively affect DNA damage signaling in T47D cells, determining by western blotting of selected important DDR factors. **(B)** Densitometric analysis of western blotting in (A).

**Figure S3. TRPS1 promotes DNA repair by enhancing NHEJ pathway in BC cells.**

**(A)** A schematic diagram shows the mechanism of EJ5SceGFP system for cell-based NHEJ assays. **(B)** A representative western blot shows that expression of TRPS1 is undetectable in U2OS cells using MCF7 as a positive reference. **(C)** A representative western blot shows that Ku70 is successfully silenced using siRNAs in MCF7 cells.

**Figure S4. TRPS1 regulated DDR depend on its non-transcription factor function.**

**(A)** mRNA expression profile analysis shows the expression of genes involved in DNA damage response in MCF7 and T47D cells TRPS1 depletion based on published RNA-seq data (GSE107023). **(B)** Expression of selected DDR genes were measured in MCF7 cells with or without expressing TRPS1 by RT-qPCR, and the result shows that depletion of TRPS1 by CRISPR-Cas9 does not affect the expression of selected DDR genes. **(C)** Expression of selected DDR genes were measured in T47D cells transducing with TRPS1 siRNAs or control by RT-qPCR, and the result shows that depletion of TRPS1 does not affect the expression of selected important DDR genes. **(D)** Expression of selected DDR genes were measured by RT-qPCR in MCF7^Par^ and MCF7^TRPS1-KO^ cells either without or with ADR treatment, DMSO treatment as control. The result shows that depletion of TRPS1 does not affect the expression of selected important DDR genes. **(E)** MCF cells were treated with 5 μM ADR or DMSO, and the impact on γH2AX abundance was quantified by western blot. A representative image of western blot shows that ADR treatment increases protein level of γH2AX but not TRPS1 in MCF7 cells. **(F)** MCF cells were challenged with 5 μM ADR for 2 hours, subsequently chased in fresh media as indicated time points. Western blot was performed with antibodies indicated. Depletion of TRPS1 can negatively affect DNA damage signaling in MCF7 cells, determining by western blotting of TRPS1. A representative image of western blot shows that TRPS1 protein level is relatively stable during the recovery period after ADR treatment. **(G)** The images of degradation with or without the Deoxyribonuclease I using MCF7 cells lysates. **(H)** Expression of Ku70 and Ku80 were diminished with siRNAs in Hela cells, and the impact on TRPS1 mRNA expression was quantified by qPCR. **(I)** Domain organization of TRPS1 and its truncation mutations. Graph bars and error bars represent respectively the mean±SD of three independent experiments. Statistics analysis was performed using one-way ANOVA. *P<0.05, **P<0.01,****P<0.0001.

**Figure S5. A direct association exists between TRPS1 and PARylation modifications.**

**(A)** HEK293T cells were transfected with truncated TRPS1 and TRPS1 full length for 72 hours, subsequently collected for lysates and immunoprecipitated with the Flag antibody. Finally, immunoblotted with the antibodies indicated. Domain mapping assays show that the GATA domain of TRPS1 is required for its association with PARP1. **(B)** Co-IP analyses of the co-immunoprecipitation relationship between TRPS1 and PARylation and Co-IP shows that TRPS1 can be precipitated by anti-PAR antibody. **(C)** MCF7 cells were treated with ADR and AG14361 for 24 hours, DMSO as control. Subsequently cell collected for lysates and immunoprecipitated with the TRPS1 antibody or control IgG. Finally, immunoblotted with the antibodies indicated. PARylation levels of TRPS1-binding proteins are increased along with increased activated PARP1 in MCF7 cells. **(D)** T47D cells were treated with ADR and AG14361 for 24 hours, DMSO as control. Subsequently cell collected for lysates and immunoprecipitated with the TRPS1 antibody or control IgG. Finally, immunoblotted with the antibodies indicated. PARylation levels of TRPS1-binding proteins are increased along with increased activated PARP1 in T47D cells. **(E)** RT-qPCR shows that PARP1 is successfully silenced by PARP1 siRNAs. **(F)** RT-qPCR shows that PARG is successfully silenced by PARG siRNAs. Graph bars and error bars represent respectively the mean±SD of three independent experiments. Statistics analysis was performed using a student t-test (two-tailed). ***P<0.001.

**Figure S6. ADR sensitivity assays to determine the ADR sensitivity of TRPS1 mutations on breast tumor cells. (A)** HEK293T cells were transfected with TRPS1 mutants and TRPS1 full length (FL) for 72 hours, subsequently collected for lysates and immunoprecipitated with the TRPS1 antibody and control IgG. Finally, immunoblotted with the antibodies indicated. **(B)** MCF7^TRPS1-KO^ cells were transfected with TRPS1 mutants and TRPS1 abundance was quantified by immunoblotting, TRPS1 full length as control. **(C)** MCF7^TRPS1-KO^ cells were transfected with TRPS1 mutants and subsequently treated with ADR as indicated concentration for 48 hours. Then cell viability was measured by CCK8 kit and the relative cell viability was calculated via compared to per TRPS1 mutant without ADR treatment. Graph bars and error bars represent respectively the mean±SD of three independent experiments. Statistics analysis was performed using a student t-test (two-tailed). *P<0.05,**P<0.01, ns, not significant.

**Dataset legend**

Dataset 1: Identification and analysis of TRPS1 binding proteins

**Supporting Information Tables**

**Table S1. Clinical and pathological characteristics of collected BC patients who received chemotherapy**

| Characteristics | TRPS1 High | TRPS1 Low | Total |
| --- | --- | --- | --- |
| Age (n=61) |  |  |  |
| ≥45year | 21 | 19 | 40 |
| <45year | 12 | 9 | 21 |
| Gender(n=61) |  |  |  |
| Female | 33 | 28 | 61 |
| Male | 0 | 0 | 0 |
| Diameter(n=61) |  |  |  |
| ≥2cm | 16 | 12 | 28 |
| <2cm | 17 | 16 | 33 |
| Primary cancer site(n=61) |  |  |  |
| Left | 12 | 16 | 28 |
| Right | 21 | 12 | 33 |
| Invasion(n=61) |  |  |  |
| Yes | 33 | 27 | 60 |
| No | 0 | 1 | 1 |
| Histological subtype(n=60) |  |  |  |
| Lobular | 2 | 0 | 2 |
| NST | 17 | 11 | 28 |
| Ductal | 14 | 16 | 30 |
| Claudin subtype(n=61) |  |  |  |
| Luminal | 27 | 13 | 40 |
| HER2 | 5 | 12 | 17 |
| Basal | 1 | 3 | 4 |
| Total Patient no.: 61 |  |  |  |
| Sample collected method |  |  |  |
| Biopsy | 36 | 24 | 60 |
| Surgery | 20 | 29 | 49 |
| #Total tumor sample no.: 109 |  |  |  |

Abbreviations: n and no., number; NST, invasive carcinoma of no special type; HER2, human epidermal growth factor receptor 2; #39 patients provided samples contained both biopsy and surgery sample (9 patients provided two biopsy samples), 22 patients provided either biopsy or surgery sample.

**Table S2. IC50 values of ADR and Etoposide in BC cells**

| Cell line | Adriamycin (μM) | Etoposide (μM) |
| --- | --- | --- |
| **Low TRPS1 expression** | |  |
| MCF-10A | 0.05±0.01 | 0.21±0.02 |
| MDA-MB-231 | 0.11±0.03 | 10.16±0.95 |
| BT549 | 0.14±0.01 | 0.89±0.25 |
| MDA-MB-468 | 0.07±0.01 | 1.66±0.25 |
| Hs578T | 0.25±0.04 | 8.80±0.77 |
| **High TRPS1 expression** | |  |
| MCF-7 | 4.89±1.76 | 10.15±0.45 |
| T47D | 0.70±0.09 | 299.90±26.53 |
| BT474 | 0.03±0.01 | 88.26±6.47 |

**Table S3.** **siRNAs used in this study**

| SiRNA | SOURCE | SEQUENCE/ IDENTIFIER |
| --- | --- | --- |
| Control siRNA sense  Control siRNA antisense | GenePharma  GenePharma | 5'-UUCUCCGAACGUGUCACGUTT-3'  5'-ACGUGACACGUUCGGAGAATT-3' |
| TRPS1 siRNA-1 sense  TRPS1 siRNA-1 antisense | GenePharma  GenePharma | 5'-GUCCCUUGAAUGUAGUAAATT-3'  5'-UUUACUACAUUCAAGGGACTT-3 |
| TRPS1 siRNA-2 sense  TRPS1 siRNA-2 antisense | GenePharma  GenePharma | 5'-GCACACAGCUGCUACAAAUTT-3'  5'-AUUUGUAGCAGCUGUGUGCTT-3' |
| PARP1 siRNA1 sense  PARP1siRNA1 antisense | GenePharma  GenePharma | 5'-GAGGAAGGUAUCAACAAAUTT-3'  5'-AUUUGUUGAUACCUUCCUCCTT-3' |
| PARP1 siRNA2  PARP1 siRNA2 antisense | GenePharma  GenePharma | 5'-GAGCACUUCAUGAAAUUAUTT-3'  5'-AUAAUUUCAUGAAGUGCUCTT-3' |
| PARG siRNA1 sense  PARG siRNA1 antisense | GenePharma  GenePharma | 5'-GCGGUGAAGUUAGAUUACATT-3'  5'-UGUAAUCUAACUUCACCGCTT-3' |
| PARG siRNA2 sense  PARG siRNA2 antisense | GenePharma  GenePharma | 5'-AAAUGGGACUUUACAGCUUUG-3'  5'-CAAAGCUGUAAAGUCCCAUUU-3' |
| Ku70 siRNA1 sense  Ku70 siRNA1 antisense | TSINGKE  TSINGKE | 5'-GUUCUAUGGUACCGAGAAA-3'  5'-UUUCUCGGUACCAUAGAAC-3' |
| Ku70 siRNA2 sense  Ku70 siRNA2 antisense | TSINGKE  TSINGKE | 5'-CGGUGAUCUCCGAGAUACA-3'  5'-UGUAUCUCGGAGAUCACCG-3' |
| Ku80 siRNA1 sense  Ku80 siRNA1 antisense | TSINGKE  TSINGKE | 5'-GCGAGUAACCAGCUCAUAA-3'  5'-UUAUGAGCUGGUUACUCGC-3' |
| Ku80 siRNA1 sense  Ku80 siRNA1 antisense | TSINGKE  TSINGKE | 5'-GCAUGGAUGUGAUUCAACA-3'  5'-UGUUGAAUCACAUCCAUGC-3' |

**Table S4.** **The primers used for construction of TRPS1 truncations**

| PRIMERs | SOURCE | SEQUENCE |
| --- | --- | --- |
| pEGFP-N2-TRPS1-F | GENEWIZ | 5'-CCGGAATTCATGCCTTATGAAGTCAATGC-3' |
| pEGFP-N2-TRPS1-R | GENEWIZ | 5'-CGGGATCCCCTCTTTAGGTTTTCCAT-3' |
| pEGFP-N2-TRPS1-C-F | GENEWIZ | 5'-CCGGAATTCATGAGGGGCAGCAATGAGGAGCAAG-3' |
| pEGFP-N2-TRPS1-C-R | GENEWIZ | 5'-CGGGATCCCCTCTTTAGGTTTTCCAT-3' |
| pEGFP-N2-TRPS1-△N-F | GENEWIZ | 5'-CCGGAATTCATGCAGCAGTATCCTGCATCGGG-3' |
| pEGFP-N2-TRPS1-△N-R | GENEWIZ | 5'-CGGGATCCCCTCTTTAGGTTTTCCAT-3' |
| pEGFP-N2-TRPS1-△C-F | GENEWIZ | 5'-CGGGATCCCCTCTTTAGGTTTTCCAT-3' |
| pEGFP-N2-TRPS1-△C-R | GENEWIZ | 5'-CGGGATCCCCTGCTGTTTGTTGAGCTGCTCA-3' |
| pEGFP-N2-TRPS1-N-F | GENEWIZ | 5'-CGGGATCCCCTCTTTAGGTTTTCCAT-3' |
| pEGFP-N2-TRPS1-N-R | GENEWIZ | 5'-CGGGATCCCGGGGAGGGCCCCAGACTTCT-3' |
| pEGFP-N2-TRPS1-GATA-F | GENEWIZ | 5'-CCGGAATTCATGCAGCAGTATCCTGCATCG-3' |
| pEGFP-N2-TRPS1-GATA-R | GENEWIZ | 5'-CGGGATCCCCTGCTGTTTGTTGAGCTGCTCA-3' |

**Table S5.** **Primers of TRPS1 mutations**

| PRIMERs | SOURCE | SEQUENCE |
| --- | --- | --- |
| TRPS1-E28A-F | GENEWIZ | 5'-CTCTGAGAAACGTTGCAAGT**GCA**  GGCGAGGGCCAGATCCTGGA-3' |
| TRPS1-E28A-R | GENEWIZ | 5'-TCCAGGATCTGGCCCTCGCC**TGC**  ACTTGCAACGTTTCTCAGAG-3' |
| TRPS1-E30A-F | GENEWIZ | 5'-GAAACGTTGCAAGTGAAGGC**GCA**  GGCCAGATCCTGGAGCCTAT-3' |
| TRPS1-E30A-R | GENEWIZ | 5'-ATAGGCTCCAGGATCTGGCC**TGC**  GCCTTCACTTGCAACGTTTC-3' |
| TRPS1-E40A-F | GENEWIZ | 5'-TCCTGGAGCCTATAGGTACA**GCA**  AGCAAGGTATCTGGAAAGAA-3' |
| TRPS1-E40A-R | GENEWIZ | 5'-TTCTTTCCAGATACCTTGCT**TGC**  TGTACCTATAGGCTCCAGGA-3' |
| TRPS1-D63A-F | GENEWIZ | 5'-CAGAAAATACGGATCAGAGT**GCA**  GCTGCAGAACTAAATCATAA-3' |
| TRPS1-D63A-R | GENEWIZ | 5'-TTATGATTTAGTTCTGCAGC**TGC**  ACTCTGATCCGTATTTTCTG-3' |
| TRPS1-E362A-F | GENEWIZ | 5'-ATATGGGCAACTCATCCACC**GCA**  TTAGAACAACATTTTCTTCA-3' |
| TRPS1-E362A-R | GENEWIZ | 5'-TGAAGAAAATGTTGTTCTAA**TGC**  GGTGGATGAGTTGCCCATAT-3' |
| TRPS1-E383A-F | GENEWIZ | 5'-AAGCTTCTCTCCCCTCCTCT**GCA**  GTTGCAAAACCTTCAGAGAA-3' |
| TRPS1-E383A-R | GENEWIZ | 5'-TTCTCTGAAGGTTTTGCAAC**TGC**  AGAGGAGGGGAGAGAAGCTT-3' |
| TRPS1-E457A-F | GENEWIZ | 5'-AATTTTGTAGTTTCAGCTGT**GCA**  TCATCTAGCTCACTTAAACT-3' |
| TRPS1-E457A-R | GENEWIZ | 5'-AGTTTAAGTGAGCTAGATGA**TGC**  ACAGCTGAAACTACAAAATT-3' |
| TRPS1-E504A-F | GENEWIZ | 5'-ATGATCTAGCCAAAAGTTCA**GCA**  GGAGAGACAATGACCAAGAC-3' |
| TRPS1-E504A-R | GENEWIZ | 5'-GTCTTGGTCATTGTCTCTCC**TGC**  TGAACTTTTGGCTAGATCAT-3' |
| TRPS1-E506A-F | GENEWIZ | 5'-TAGCCAAAAGTTCAGAAGGA**GCA**  ACAATGACCAAGACAGACAA-3' |
| TRPS1-E506A-R | GENEWIZ | 5'-TTGTCTGTCTTGGTCATTGT**TGC**  TCCTTCTGAACTTTTGGCTA-3' |
| TRPS1-E775A-F | GENEWIZ | 5'-AAATGGGAGAGCCAGTTTCT**GCA**  AGTGTGGTGAAGAGAGAGAA-3' |
| TRPS1-E775A-R | GENEWIZ | 5'-TTCTCTCTCTTCACCACACT**TGC**  AGAAACTGGCTCTCCCATTT-3' |
| TRPS1-E847A-F | GENEWIZ | 5'-TAAGGGATAGTCCCAATGTG**GCA**  GCCGCCCATCTGGCGCGACC-3' |
| TRPS1-E847A-R | GENEWIZ | 5'-GGTCGCGCCAGATGGGCGGC**TGC**  CACATTGGGACTATCCCTTA-3' |
| TRPS1-E1057A-F | GENEWIZ | 5'-TTCAGATAAAAAGTCCTCAG**GCA**  AGTACTGGAGATCCAGGAAA-3' |
| TRPS1-E1057A-R | GENEWIZ | 5'-TTTCCTGGATCTCCAGTACT**TGC**  CTGAGGACTTTTTATCTGAA-3' |
| TRPS1-E1070A-F | GENEWIZ | 5'-GAAATAGTTCATCCGTATCT**GCA**  GGGAAAGGAAGTTCTGAGAG-3' |
| TRPS1-E1070A-R | GENEWIZ | 5'-CTCTCAGAACTTCCTTTCCC**TGC**  AGATACGGATGAACTATTTC-3' |
| TRPS1-E1225A-F | GENEWIZ | 5'-TTGATAGAAGTACTCAAGAT**GCA**  CTTTCAACAAAATGTGTGCA-3' |
| TRPS1-E1225A-R | GENEWIZ | 5'-TGCACACATTTTGTTGAAAG**TGC**  ATCTTGAGTACTTCTATCAA-3' |
| TRPS1-E1287A-F | GENEWIZ | 5'-ATAGGAACAATGCACAAGTG**GCA**  AAAAATGGAAAACCTAAAGA-3' |
| TRPS1-E1287A-R | GENEWIZ | 5'-TCTTTAGGTTTTCCATTTTT**TGC**  CACTTGTGCATTGTTCCTAT-3' |

**Table S6.** **Primers for quantitative PCR**

| PRIMERs | SOURCE | SEQUENCE |
| --- | --- | --- |
| H2AFX qpcr F | GENEWIZ | 5'-AACGACGAGGAGCTCAACAAGC-3' |
| H2AFX qpcr R | GENEWIZ | 5'-TGGCGCTGCTCTTCTTGGGCA-3' |
| PARP1 qpcr F | GENEWIZ | 5'-CGGAGTCTTCGGATAAGCTCT-3' |
| PARP1 qpcr R | GENEWIZ | 5'-TTTCCATCAAACATGGGCGAC-3' |
| Ku70 qpcr F | GENEWIZ | 5'-TCATGGCAACTCCAGAGCAG-3' |
| Ku70 qpcr R | GENEWIZ | 5'-AACCTTGGGCAATGTCAGGT-3' |
| Ku80 qpcr F | GENEWIZ | 5'-CCATGAGCTTGGCAAAGAAAG-3' |
| Ku80 qpcr R | GENEWIZ | 5'-GTGCAGCAGACACTGAAATAATC-3' |
| 53BP1 qpcr F | GENEWIZ | 5'-AAGCCAGGCAAGAGAATGAGGC-3' |
| 53BP1 qpcr R | GENEWIZ | 5'-GGCTGTTGACTCTGCCTGATTG-3' |
| BRCA1 qpcr F | GENEWIZ | 5'-GAAACCGTGCCAAAAGACTTC-3' |
| BRCA1 qpcr R | GENEWIZ | 5'-CCAAGGTTAGAGAGTTGGACAC-3' |
| ATM qpcr F | GENEWIZ | 5'-TGTTCCAGGACACGAAGGGAGA-3' |
| ATM qpcr R | GENEWIZ | 5'-CAGGGTTCTCAGCACTATGGGA-3' |
| ATR qpcr F | GENEWIZ | 5'-GGAGATTTCCTGAGCATGTTCGG-3' |
| ATR qpcr R | GENEWIZ | 5'-GGCTTCTTTACTCCAGACCAATC-3' |
| CHK2 qpcr F | GENEWIZ | 5'-GACCAAGAACCTGAGGAGCCTA-3' |
| CHK2 qpcr R | GENEWIZ | 5'-GGATCAGATGACAGCAGGAGTTC-3' |
| CHK1 qpcr F | GENEWIZ | 5'-GTGTCAGAGTCTCCCAGTGGAT-3' |
| CHK1 qpcr R | GENEWIZ | 5'-GTTCTGGCTGAGAACTGGAGTAC-3' |
| TP53 qpcr F | GENEWIZ | 5'-CCTCAGCATCTTATCCGAGTGG-3' |
| TP53 qpcr R | GENEWIZ | 5'-TGGATGGTGGTACAGTCAGAGC-3' |
| LIG4 qpcr F | GENEWIZ | 5'-CAGCAGAGATCGTACCCAGTGA-3' |
| LIG4 qpcr R | GENEWIZ | 5'-TGCGAGCTTACCAGATGCCTTC-3' |
| XRCC4 qpcr F | GENEWIZ | 5'-ATGGCTCCTCAGGAGAATCAGC-3' |
| XRCC4 qpcr R | GENEWIZ | 5'-GAGGTCTTCTGGGCTGCTGTTT-3' |
| PARG qpcr F | GENEWIZ | 5'-CCAGTTGGATGGACACTAAAGG-3' |
| PARG qpcr R | GENEWIZ | 5'-GCAGTCTGATGCTGGTTCAAA-3' |
| Actin qpcr F | GENEWIZ | 5'-AACCCTAAGGCCAACCGTGA-3' |
| Actin qpcr R | GENEWIZ | 5'-GTCTCCGGAGTCCATCACAA-3' |
| TRPS1 qpcr F | GENEWIZ | 5'-TCTACCAGAAGCTTCACTCG-3' |
| TRPS1 qpcr R | GENEWIZ | 5'-CTCTCTAACGGGCTTCCATT-3' |

**Table S7.** **Antibodies for western blotting**

| Antibodies | | |
| --- | --- | --- |
| TRPS1 | R&D Systems | Cat# AF4838 |
| Anti-TRPS1 | Abcam | Cat# ab209664 |
| β-actin | ProteinTech | Cat# 60008-1-lg |
| Phospho-ATM -S1981 | ABclonal | Cat# AP0008 |
| ATM | ABclonal | Cat# A5908 |
| Phospho-Chk2(Thr68) | Affinity | Cat# AF3036 |
| Chk2 | ProteinTech | Cat# 13954-1-AP |
| Phospho-H2A.X (Ser139) | Sigma-Aldrich | Cat# 05-636 |
| Anti-gamma H2A.X (phospho S139) | Abcam | Cat# ab81299 |
| PARP1 | ABclonal | Cat# A0942 |
| Ku70 | ABclonal | Cat# A7330 |
| Ku80 | ABclonal | Cat# A5862 |
| XRCC4 | ProteinTech | Cat#15817-1-AP |
| DNA-PKcs | Santa Cruz | Cat#SC-5282 |
| LIG4 | ProteinTech | Cat#12695-1-AP |
| HRP Goat Anti-Rabbit IgG | ABclonal | Cat# AS014 |
| Rabbit anti DDDDK-Tag pAb | ABclonal | Cat# AE004 |
| Donkey anti-goat IgG-HRP | Santa Cruz | Cat# SC-2020 |
| Anti-PAR Polyclonal | R&D Systems | Cat# 4336-APC-050 |
| OctA (DYKDDDDK)-Probe | Santa Cruz | Cat# SC-807-G |
